# Why do autocracies enfranchise their citizens abroad? A large-N event history analysis, 1990–2010

**DOI:** 10.1080/13510347.2024.2383795

**Published:** 2024-08-22

**Authors:** Nicolas Fliess, Ali Kiani, Eva Østergaard-Nielsen

**Affiliations:** a Department of Socio-Cultural Diversity, Max Planck Institute for the Study of Religious and Ethnic Diversity, Goettingen, Germany; b Independent Researcher; c Department of Political Science and Public Law, Autonomous University of Barcelona, Barcelona, Spain

**Keywords:** Diaspora, autocracies, external voting, enfranchisement, elections

## Abstract

Autocratic ruling elites allow elections as a survival strategy. Many authoritarian regimes have taken this tactic one step further, also inviting their diaspora to vote from afar. This may seem puzzling given that elections abroad are difficult to control and provide a platform for exiled regime critics. So far, however, the reasons for autocracies to grant their diaspora voting rights have rarely been explored. In this article, we address this shortcoming. We employ a cross-national, autocratic regime dataset and a discrete-time event history model. We argue that autocracies use diaspora suffrage to repress and co-opt their citizens abroad while legitimizing their rule domestically and internationally. Autocrats are risk-averse and the decision to enfranchise hinges on the characteristics of the diaspora and the regime’s need for legitimacy, repression and co-optation after power transitions. We substantiate these claims by demonstrating that autocrats are less likely to enfranchise a diaspora that largely resides in democracies or consists of refugees in democracies. In turn, successful coups render diaspora suffrage adoption more probable. In sum, external voting rights present a critical case to better understand why authoritarian states adopt democratic institutions and wish to connect with their internationally mobile population.

## Introduction

In recent decades numerous states have granted voting rights to their citizens abroad. As of today, 141 countries have adopted voting laws that allow emigrants to vote in homeland elections, a significant increase from the mere 38 countries in 1990.^1^ At a first glance, one could assume that external voting rights would be limited to consolidated and developing democracies, since autocrats might have an interest in withholding such an important mouthpiece from citizens that have deserted authoritarian rule. In comparison with political activists inside the country, diaspora groups can publicly voice their opposition with greater ease when they reside in democratic host countries.^2^ Even so, by 2010, half of all autocracies worldwide had granted external voting rights to their emigrants. By locating this puzzle in both the broader understanding of homeland-diaspora relations and liberal institutions in autocracies, we identify a series of factors that help unpack why non-democratic states seek to seemingly democratize transnational relations with citizens abroad. We argue that autocracies extend voting rights to repress and co-opt their citizens abroad while legitimizing their rule domestically and internationally. Authoritarian regimes carefully balance the risks and benefits of granting voting rights to emigrants by considering the characteristics of the diaspora and the regime’s need for repression, co-optation and legitimacy after power transitions.

To test these arguments, we break new ground by analysing enfranchisement processes across 88 autocracies between 1990 and 2010. Using a cross-national, autocratic regime dataset we study the motivations in relation to the challenges that autocracies face when granting voting rights to emigrants. First, we show that a large diaspora or refugee population in democratic host countries tempers autocracies’ willingness to adopt emigrant voting rights as this diaspora profile is associated with potential risks. Second, we show that autocracies who have recently experienced a successful coup d’état extend voting rights to their diaspora in response to a more acute need for co-optation and legitimization at home and abroad. In this scenario, diaspora suffrage adoption is used to co-opt key actors during electoral processes and signal to the citizens abroad that the new regime values them as part of the polity. It can also serve as a tool to repress and collect information abroad, while demonstrating to the international community that the coup plotters aspire to adhere to democratic principles. We conduct a range of robustness tests to confirm the results.

This article contributes to the broader understanding of how autocracies navigate the challenges posed by mobile citizenry and respond to both international and domestic pressures for democratization and legitimacy in an increasingly globalized world. Such focus adds to several ongoing debates across both the study of autocracies and the rapidly growing research field on homeland-diaspora relations. First, we expand previous work on the adoption of nominally democratic institutions by authoritarian states. A wealth of studies highlights how autocracies have introduced constitutions,^3^ political parties, elections, and legislatures,^4^ including women quotas,^5^ as a survival strategy.^6^ However, this literature has largely ignored the extra-territorial dimension of electoral politics in autocratic states. Examining diaspora voting rights in authoritarian regimes offers an important lens into how autocracies approach, court and instrumentalize their citizen abroad.

Second, we advance the literature on sending states and homeland-diaspora relations by strengthening the dialogue between this research field and the broader literature on autocracies.^7^ In so doing, we address gaps within two main strands of this literature. First, a recent addition to the general literature on outreach policies of sending countries focuses on the ways in which autocratic countries of origin seek to govern and control their citizens abroad.^8^ However, this literature has so far only paid scant attention to the question of voting rights. In the other strand, much scholarly attention has been devoted to the question of why states grant electoral rights to emigrants,^9^ but authoritarian regimes have received little consideration in this debate. Several important works have presented in-depth case studies from mainly the Middle East and North Africa.^10^ Yet the lack of broader systematic studies leaves the essential question of whether enfranchisement processes in autocracies and democracies follow similar logics largely unanswered. We demonstrate that external voting rights serve as a critical case to better understand how and why authoritarian states adopt seemingly democratic institutions to connect with the part of the polity that resides abroad.

## Autocracies, voting rights and emigration in a transnational perspective

Emigrant voting rights can broadly be defined as the right to vote by citizens who reside outside their country of citizenship. We focus on the legislation adopted to permit non-resident citizens to cast their ballot while residing in another country. Emigrant voting rights are not new. Already, in the early twentieth century, some countries offered postal voting to diplomatic staff, soldiers, and seafarers who were abroad on election day. Since the 1950s, states started pursuing a more inclusive approach, extending voting rights to citizens beyond narrowly defined professions. Notably, the authoritarian regime of Indonesia led the way as an early enfranchiser in 1953.^11^

**Figure 1.**
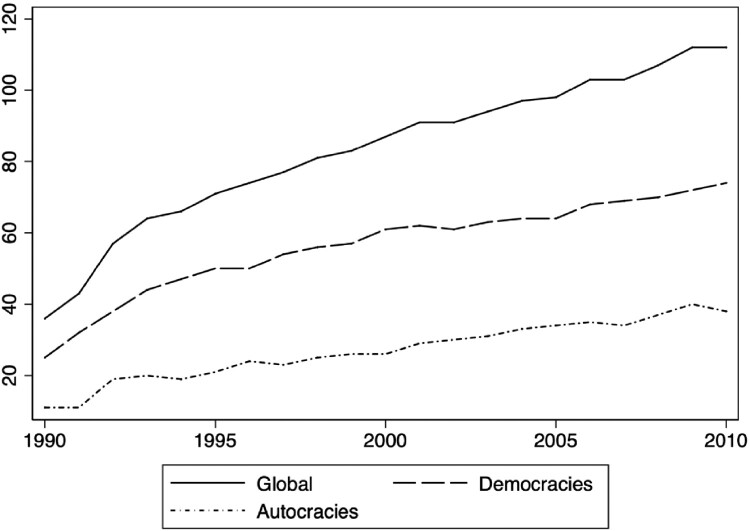
External voting by regime type. Note: the figure shows the number of states with *de jure* emigrant voting rights by regime type over the period 1990–2010. Information on emigrant enfranchisement is from Wellman, Allen, and Nyblade (2023). Regime type categorization is based on Boix, Miller, and Rosato (2013).

In this article, we mainly concentrate on the introduction of *de jure* voting rights for emigrants on the national level. While external voting rights have become a standard practice in most democracies, authoritarian regimes are only slightly lagging behind. Figure 1 shows the global enfranchisement trend by regime type for the 1990–2010 period. By 2010, half of all autocracies worldwide had granted diaspora voting rights and over three-fourths of them also held *de facto* elections abroad.

In line with the global expansion of emigrant voting rights, research on states’ motivations to grant them has flourished since the mid-2000.^12^ These studies emphasize global norm diffusion processes,^13^ government motivations to secure resources abroad,^14^ party competition dynamics,^15^ and regime transitions to democratic rule.^16^ Especially, the driving force of transitions to democracy cannot be used to explain the many autocracies who have enfranchised their diaspora.

## Emigrant enfranchisement in autocracies: a strategy for repression, legitimation and co-optation of the diaspora

Emigration plays a central role in many authoritarian regimes. Traditionally, authoritarian leaders have constrained emigration in order to increase regime stability.^17^ Yet, nowadays authoritarian states have identified emigrants as a valuable source of revenue and legitimization.^18^ This shift in perspective has gone hand in hand with a stronger engagement in extra-territorial policy making, adopted to bolster authoritarian resilience.^19^ Autocratic states find themselves in an “illiberal paradox”^20^ having to balance the economic need for open borders with their continued desire to control their citizens.

Drawing on Gerschewski’s typology of strategies for autocratic stability comprising repression, legitimation and co-optation,^21^ recent studies argue that autocratic sending countries also apply these three strategies in their relations with citizens abroad.^22^ Repression includes the surveillance and persecution of dissidents abroad.^23^ Autocracies can use visa and passport procedures to control the opposition (earlier referred to as “long-distance policing”),^24^ punish homeland-based family members of emigrants,^25^ de-nationalize undesired individuals and even attack, abduct or assassinate their citizens abroad.^26^ Legitimation strategies include attempts to foment loyalty among citizens through discourses or activities conveying the importance and belonging of citizens abroad in their homeland, somewhat akin to the diaspora building concept of Gamlen.^27^ Finally, co-optation turns citizens abroad into clients.^28^ In the framework of Gerschewski,^29^ this strategy relates to the co-optation of elite actors, but in the transnational dimension it can target a broader segment of the diaspora. An example is patronage through offering a range of benefits to those diaspora members who are helping from afar,^30^ such as the doubling of pensions for those Crimeans who retain their Russian passport.^31^

Curiously, voting rights are largely omitted from these recent analyses of autocratic transnational strategies. Yet, many authoritarian regimes periodically organize elections. The key reasons for doing so echo the typology of Gerschewski^32^: elections may improve the capacity of regimes to repress the opposition^33^ by demonstrating that they can effectively control large-scale mobilizations, such as electoral contests.^34^ Elections also help increase the regime’s legitimacy^35^ and co-opt key actors through an improved ability to distribute rents and extend patronage.^36^ Against this background, holding elections may seem like a low-risk high-gain strategy in so far that authoritarian regimes have the resources and power to tilt the electoral playing field in their favour.^37^

Granting external voting rights, we argue, follows the same logic. In terms of repression, extending voting rights to overseas citizens can provide autocracies with a powerful tool to gain information on them which in turn facilitates repression.^38^ While regimes are likely to be aware of the activities of high-profile exiles, elections can provide information about broader patterns of overseas dissidence. Voter registers at consulates or online can be used to collect sensitive personal information among supporters and dissidents. Moreover, public campaign events in countries of residence render support for the opposition more visible. Finally, online surveillance on social media and opposition websites can be better targeted before and after elections.^39^

In terms of legitimation, the extension of voting rights can help forge loyalty among citizens abroad. Allowing overseas citizens to become part of the polity from afar sends a strong message of inclusion.^40^ Emigrants may hold the homeland regime responsible for the economic and political conditions causing their departure. Yet, voting rights may serve as an olive branch whereby the homeland government recognizes their continued membership. Indeed, previous work on emigrant suffrage in democracies has found that emigrants tend to vote for the party who has enfranchised them.^41^ Extending voting rights may not work as a legitimizing strategy for the core exile opposition abroad, yet it may have an impact on the broader emigrant collective.^42^

In terms of co-optation, voting rights can signal increased attention to diaspora-specific demands. The list of such demands is usually long, including extended consular services, opportunities for investment and the avoidance of military service.^43^ Voting rights can also be a way of co-opting prominent opposition leaders. Countries such as Algeria, Guinea-Bissau or Mauritania maintain designated seats in their parliament for members of the diaspora who are elected abroad during national elections.^44^ Research in partly free democracies demonstrates that parties often co-opt the overseas leaders of prominent migrant organizations by nominating them as candidates for these seats.^45^ Authoritarian regime parties can thus use extra-territorial parliamentary seats to extend patronage abroad.

With these general incentives in mind, we turn to two sets of explanations for why autocracies do and do not extend voting rights to their citizens abroad. Specifically, we focus on the risks and benefits that autocracies associate with diaspora suffrage. First, the level of democracy in the residence countries and the share of refugees among citizens abroad may temper the extension of voting rights abroad. Second, domestic political developments (power transitions and coups) in autocracies may drive the extension of voting rights.

### The democratic destination effect

A particular characteristic influencing state-diaspora relations is the diaspora’s place of residence. Betts and Jones argue that in authoritarian countries “diaspora politics has its greatest geopolitical impact when it is directed at leveraging the foreign policies of liberal democratic states.”^46^ Authoritarian home states enact economic, political, social, religious, and cultural policies to repress, legitimize and co-opt diasporas in democratic destinations so that they can become their allies in influencing the policy affairs of their countries of residence.^47^ This policy outreach package may also include voting rights and we argue that the political regimes hosting the diaspora shape the decision to extend them.

First, holding elections abroad may affect bilateral relations because it requires collaboration with the government of the jurisdiction where the election takes place. Host countries not only provide infrastructure beyond the diplomatic missions to hold, organize and coordinate electoral processes, but also issue travel documents for election staff and monitors, clear customs for election materials, assist with security provisions and provide demographic data on voters.^48^ However, host country governments can also refuse to collaborate over concerns about the election’s integrity, security and moral implications. Diaspora groups might amplify these integrity concerns. The overseas Congolese electorate, for instance, petitioned the governments of France, South Africa and the United States to publicize the number of Congolese nationals to reduce the risk of voter fraud and increase voter participation.^49^ Enough pressure from abroad can even force homeland authorities to make concessions. For example, in 2011, Cameroon’s electoral commission representatives travelled abroad to meet with the diaspora who rallied to boycott the voting over election integrity concerns.^50^ In terms of security, especially in highly conflictual contexts, governments may fear that elections exacerbate conflicts between different foreign actors within the country. In democracies, these concerns can be intertwined with moral concerns about supporting autocrats seeking to legitimize their rule. In the 2014 Syrian presidential elections, several Western countries prohibited out-of-country-voting on their territory.^51^ Similarly, during the 2017 Turkish constitutional referendum German, Dutch, Danish and Swiss governments cancelled or restricted pro-regime campaign activities on their soil. Such measures can strain diplomatic relationships and cast a negative light on the sending state government.

Second, destination country regimes determine the degree of freedom of oppositional organization abroad. While autocratic host-states restrict the political space, democracies offer greater freedom of speech and association to voice criticism towards the homeland regime.^52^ Hence, oppositional forces might call unwelcome attention to the lack of democracy in the country of origin. Diaspora collectives and transnational party branches based in democracies also gain a campaign funding advantage by conducting fund-raisers in strong currencies,^53^ which can help challenge the traditional political homeland elite.^54^

Finally, a significant body of research argues that a more democratic residency country correlates with a more pro-democratic outlook of migrants.^55^ In continuation, migrants diffuse their attitudes, behaviour and values through cross-border networks to the country of origin.^56^ Their economic remittances have been linked to support for opposition parties and participation in anti-government protests within the country.^57^ Unsurprisingly, authoritarian states actively control emigration to democracies to prevent such democratization effects.^58^

Voting rights are per default extended to all members of the diaspora, including both those in opposition and those loyal to the homeland regime. However, autocratic regimes can curb the impact of emigrant votes through cumbersome voting methods^59^ or transnational gerrymandering^60^ and “postpone” elections indefinitely until they are confident to win.^61^ Registration number thresholds can result in the disenfranchisement of registered voters, as seen in past Yemeni elections.^62^ Authoritarian regimes can also manipulate ballots coming from abroad. For example, in 2019, incumbent authorities in Mozambique were accused of rigging diaspora ballot boxes.^63^ Similarly, in the 2004 Afghani elections, several ballot boxes from abroad were excluded due to ballot stuffing.^64^ Finally, authoritarian regimes can also ban diaspora-backed candidates. However, rigged elections with a largely contained opposition still pose risks, particularly when the regime conducts elections for the first time. Electoral uncertainty is heightened, and the regime may struggle to effectively undermine the organized efforts of the opposition.^65^ Authoritarian leaders are also prone to electoral losses, especially when incumbents are weak and the opposition effectively organizes.^66^ Arguably, effective diaspora mobilization is more likely to occur in democratic destinations.

In sum, democratic host countries undermine the capacity of autocratic sending countries to contain potentially regime-threatening consequences arising from an enfranchised diaspora. We therefore argue that autocracies prefer safety over potential benefits.

H1: A large share of the diaspora residing in democratic countries of residence *decreases* the likelihood of an authoritarian regime to adopt emigrant voting rights.

It could be argued that autocracies having a larger share of citizens living in democracies reflect a more politically motivated migration compared to migration to autocracies. We unpack this notion in the next section where we explore the relevance of shares of refugees in democratic destinations.

### The hostile diaspora effect

The type of migration that dominates the diaspora profile is an additional factor in the cost–benefit calculation of authoritarian regimes. Autocracies produce forced migration, as dissidents or persecuted minorities whose rights and welfare are not secured by the regime seek protection in other countries. Enfranchising these populations can be risky as exile leaders may have been able to rally and organize opposition abroad by the time voting rights are extended. For example, the Zimbabwean opposition has developed a track-record of recruiting asylum seekers for its party organization in South Africa and continuously pressures the Zimbabwean regime to grant external voting rights.^67^ Finally, enfranchising refugees can send the wrong signal to host country governments who may link refugees’ electoral participation in the homeland with the end of their asylum.^68^ In continuation, authoritarian states would have to welcome back political dissidents they were glad to see gone.^69^

A particular critical scenario emerges when a large share of citizens abroad are refugees who live in democracies. This is because of the supposed protection of their right to free speech and association in democratic host countries. In the opposite scenario, forced migrants in autocratic states often do not have political asylum and instead depend on precarious work-contracts for their residence. In the case of Eritreans in the Gulf and Sudan this situation renders them vulnerable to the demands of transnational homeland institutions.^70^ Diasporas in non-democratic countries face restricted political space, as their authoritarian hosts typically forbid political activities and may not protect them from the homeland regime.^71^

This lack of protection in non-democratic countries makes it easier for authoritarian regime actors to reach, repress and co-opt potential voters with refugee or asylum seeker status. For example, in the 1997 Liberian Election, incumbent president Taylor reportedly had busses sent to transport refugees from Guinea to voting stations in Liberia, offering bribes and the promise of ending the war.^72^ Similarly, during the 2014 Syrian elections, the regime reportedly visited refugee camps in Lebanon, threatening it would document those who did not vote.^73^ In sum, emigrant enfranchisement in a scenario with a large share of refugees residing in autocracies constitutes a low risk whereas a large share residing in democracies is a high risk. We therefore hypothesize:

H2: A large share of the diaspora comprises refugees residing in democratic countries *decreases* the likelihood of an authoritarian regime to adopt emigrant voting rights.

### The effect of power transitions and successful coups

While the previously discussed diaspora profile characteristics mainly point to the risks that autocrats may face when enfranchising their diaspora, the second set of explanations emphasizes the benefits of doing so. These explanations centre on domestic political developments which are important for understanding the timing of autocratic diaspora enfranchisement. The strategic adoption of democratic institutions in the name of regime survival is rooted within the broader autocracy and democratization literature.^74^ In the aftermath of political transitions, power-seizing groups often seek ways to cement their rule. First, they engage in institution-building to improve their repression and coercion capacity.^75^ In line with our previous argument a power transition arguably increases the likelihood of emigrant enfranchisement in autocracies so that the regime can extend repression efforts beyond its borders. Second, gaining power reinforces the need to solidify existing alliances and forge new ones. Granting policy concessions is a commonly practiced strategy by autocratic coalitions that have assumed power to gain additional support.^76^ Within this process, emigrant voting rights can serve to integrate emigrant constituencies as well as domestic civil society groups supportive of such rights.

Importantly, new authoritarian regimes often must further legitimize their rule vis-à-vis the international community. They adopt specific policies to signal their commitment to democracy and reinforce their standing abroad. The goal is to maintain economic and political support from abroad in the form of, for example, trade ties, military alliances, membership in international governmental organizations and financial aid.^77^ This perspective emphasizes the potential international legitimization effect that transnational elections can provide for an authoritarian regime. Like the organization of domestic elections,^78^ transnational elections can signal the strength, confidence and commitment to democratic principles of autocratic regimes. We therefore argue:

H3: A general power transition *increases* the likelihood of an authoritarian regime to adopt emigrant voting rights.

Irregular transitions of executive power are a firm feature of autocracies. Arguably, they require more legitimization than does winning an election.^79^ Notably, military leaders who gain power through a coup frequently resort to framing their illegal removal of the incumbent as a necessary step to restore democracy.^80^ Thyne and Powell argue that both coup plotters and coup attempt defeaters have high incentives to democratize in order to (re-)gain legitimacy, prevent subsequent coups, and to maintain foreign investment levels.^81^ For example, Bahrain extended voting rights to the diaspora after a bloodless coup in 2002.^82^ That same year, Bahrain held general elections, the first ones since 1973. Similarly, the Egyptian regime used external voting rights to deepen its relationship with the diaspora after the military coup in 2013, including significant efforts to mobilize voters abroad.^83^

While both successful and failed coups threaten a regimes’ legitimacy, and increase incentives for the political elites to introduce diaspora voting rights to strengthen or regain its legitimacy, there are important differences to be noted. Successful coup plotters have a larger need to uncover and repress dissidence, legitimize their unlawful power grab in front of overseas citizens and co-opt important overseas actors as opposed to a ruling elite who successfully defeated an unlawful power seizure attempt. Hence, we hypothesize:

H4: A successful coup *increases* the likelihood of an authoritarian regime to adopt emigrant voting rights.

The general understanding of enfranchisement needs to be located in the interplay between the diaspora profile and power shifts in the homeland. This is because the pursuit of international legitimization is a central driver for authoritarian regimes to enfranchise their non-resident citizens in the aftermath of a successful coup. Their reluctance to enfranchise overseas citizens largely living in democratic countries may be overridden by the need to signal their democratic commitment to important foreign democratic powers such as the US and EU. In this view, the perceived gains outweigh the associated risks. Diaspora enfranchisement as a strategy simultaneously accomplishes several domestic and foreign policy goals: repress dissident, legitimize the regime and co-opt the diaspora, all while sending a strong and visible message regarding their willingness to introduce democratic institutions to the governments of their countries of residence.

H4.1: A successful coup *increases* the likelihood of an authoritarian regime to adopt emigrant voting rights even when the diaspora resides in democratic countries of residence.

Added to this mechanism, autocratic leaders seizing power might leverage the moment to reconnect with exiled citizens, hoping their dislike for the previous regime does not apply to the new rulers. By extending political rights to the diaspora, the new regime can strengthen its ties with them, applying the previously discussed co-optation and legitimization strategies. Considering the importance authoritarian sending states attach to the part of their diaspora who resides in democratic host countries, and the previously discussed imminent need to connect with the diaspora after a successful coup we hypothesize that:

H4.2: A successful coup *increases* the likelihood of an authoritarian regime to adopt emigrant voting rights even when a large share of the country’s refugees resides in democratic countries of residence.

## Methodology

### Research design and estimation strategy

To analyse emigrant enfranchisement patterns in autocracies we conduct a time-series cross-national analysis. Our unit of analysis is country-year. We restrict our analysis to the period 1990–2010 for which the data of our main independent variables is available. During this time emigrant enfranchisement has also gained in popularity across the globe, which makes it an interesting period for our analysis. We restrict our sample to autocracies, which we define based on the binary democracy-autocracy measure by Boix and colleagues.^84^ For robustness tests, we replicate all main findings using the V-Dem dataset to inform our case selection and related variables.^85^

Country-year observations are included in the analysis as long as two criteria are met. First, the country has not enfranchised the diaspora yet. Second, the country is an autocracy. We also include non-electoral autocracies because the regime might introduce elections together with diaspora voting rights, as it was the case in 2002 in Bahrain. In total, the dataset contains 1008 country-year observations across 88 countries of which 41 enfranchise their diaspora during the study period. We use a semi-parametric Cox regression model, appropriate to analyse discrete events across time where explanatory factors can both change over time and remain constant.^86^ Cox proportional hazard models are commonly used in event history analysis, such as policy adoption, including migrant-sending state policies.^87^ The data structure requires standard errors clustered by country.

### Operationalization

The main dependent variable is *de jure* emigrant enfranchisement. We code this variable 0 as long as the diaspora is legally disenfranchised and 1 once the diaspora has gained voting rights, based on the Emigrant Voting Rights and Restrictions (EVRR) dataset by Wellman and colleagues.^88^ If an autocracy enfranchises its diaspora, it leaves the analysis thereafter. We limit our analysis to the factors that influence the very first *de jure* enfranchisement in a given country. A minimal condition is that this right is granted to a broad part of the citizenry abroad and not only to state officials or specific professions. Additionally, we explore whether dynamics change when we consider the *de facto* organizing of elections abroad as our dependent variable.

We operationalize our main independent variables as follows. The share of the diaspora living in democratic countries is included as a logged share. To build this variable, we use the United Nations database which provides the number of emigrants for each country of origin disaggregated by country of residence.^89^ Since this data is only available in five-year intervals (1990, 1995, 2000, 2005, 2010) we interpolate missing values using the previous year. We combine this data with the Regimes of the World (RoW) typology by Coppedge and colleagues which categorizes the diaspora’s destination countries as either a democracy or an autocracy so that we can generate the share of the diaspora that resides in a democratic country.^90^ The share of the refugee population living in democratic countries is also included as a logged share. Information on the size of the refugee population by origin and destination country is derived from the United Nations High Commissioner for Refugees (UNHCR).^91^ We combine this data with the RoW typology and aggregate the numbers of refugees under UNHCR’s mandate by country of origin and the regime type of the destination countries.

General power transitions are based on the *Power Change* measure from the Autocratic Ruling Parties Dataset (ARPD).^92^ This dataset not only records larger regime transitions but also alternations of power that are often overlooked by other regime datasets.^93^ This variable is coded 1 when another ruling party or non-party regime takes office, and 0 otherwise. Power transitions through a successful coup d’état are based on the coup d’état events dataset.^94^ The authors define a coup as “a forceful seizure of executive authority and office by a dissident/opposition faction within the country’s ruling or political elites.” A coup is successful if the new executive exercises effective authority for at least one month.^95^ The variable is coded 1 in the event of a coup, and 0 otherwise. We also use the Global Instances of Coups dataset from Powell and Thyne to successfully run robustness tests.^96^ Power transitions and coups are lagged by one year.

Finally, the models control for a range of potential cofounders. The regime’s military strength, natural resources and various population characteristics can influence authoritarian resilience and are thus expected to shape the regimes’ likelihood to adopt democratic institutions, such as emigrant voting rights.^97^ We account for this by including the Composite Indicator of National Capability (CINC) from the National Material Capabilities dataset as a control variable,^98^ which we lag by one year. CINC is an aggregate index that measures state power based on six indicators: military expenditure, military personnel, energy consumption, iron and steel production, urban population, and total population. Additionally, research shows that a lower electoral management capacity decreases the likelihood of emigrant enfranchisement.^99^ We include the *v2elembcap* variable from V-Dem that measures the resource and staff capacity of national electoral management bodies (EMB) and we lag it by one year.^100^ All models also include the logged real GDP per capita,^101^ the real GDP growth,^102^ both lagged by one year. Regime age also may influence the willingness of autocrats to undertake risky electoral reforms.^103^ This continuous variable is based on the ARPD and is included as number of years.^104^ Transnational grass-root groups can pressure the home state for electoral reforms by building alliances with civil society groups inside the country.^105^ Civil society strength is based the *v2x_cspart* variable from V-Dem^106^ and included as a five-year rolling average to gauge how social capital is built up over time.^107^

Several international factors might also influence autocrats’ willingness to enfranchise emigrants. The autocratic resilience literature underscores the importance of foreign aid shaping the decision making of autocratic leaders.^108^ We therefore account for a state’s dependency on official development aid, measured in constant US dollars as a logged share of the country’s real GDP, lagged by one year.^109^ Studies on external voting rights argue that the rapid expansion of such rights relates to patterns of global and regional norms diffusion.^110^ Research on policy diffusion in autocracies suggests that this mechanism could also be relevant in non-democracies.^111^ We therefore include the *cap_delta_evrr1* variable from the EVRR dataset, which assigns 1 if one of a country’s six neighbours has introduced *de jure* emigrant voting for the first time within the previous two years, and otherwise 0.^112^ Neighboorhood effects are accounted for with the regional average of the *v2x_polyarchy* V-Dem electoral democracy score, excluding the country in question.^113^ Regions are defined based on Miller,^114^ and by using the *e_regionpol* variable from V-Dem.^115^ This variable also controls that enfranchisement processes are not embedded in larger regional democratization processes. Finally, we control for past democratic spells by creating a rolling average of democratic years over the past decade.

## Findings and discussion

**Figure 2.**
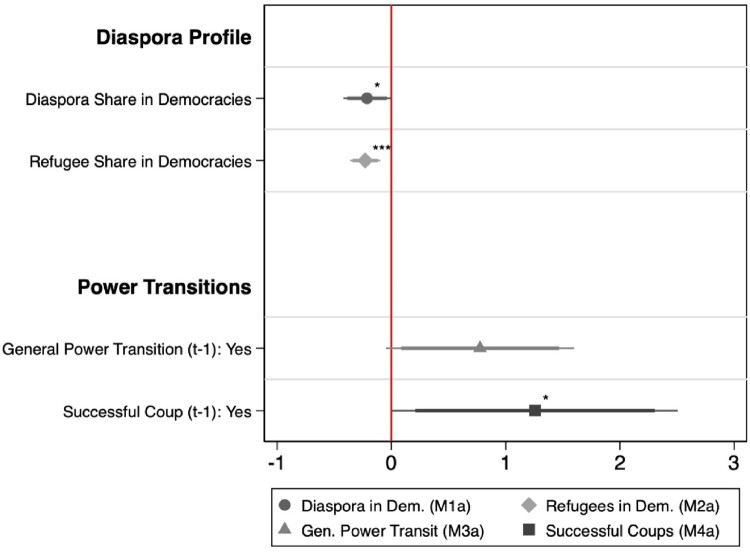
Cox regression coefficients related to the adoption of emigrant voting rights. Note: Coefficient estimates from Cox regressions with the thick bar showing the corresponding 90% confidence interval and the thin bar showing the corresponding 95% confidence interval. Dependent Variable is emigrant enfranchisement. p-values shown alongside markers: * p<0.05, ** p<0.01, *** p<0.001. All models include: state capacity (t-1), EMB capacity (t-1), GDP per capita (t-1), GDP per capita growth (t-1), time in power, civil society strength, foreign aid dependency (t-1), emigrant suffrage diffusion, regional democracy score, democratic past.

We run four separate models (M1–M4) to test the impact of (1) a large diaspora in democratic host countries, (2) a large refugee population in democratic host countries, (3) general power transitions, and (4) successful coups on diaspora enfranchisement in authoritarian regimes (H1–H4). Each model includes one of the four explanatory variables along with all the controls. The findings from the regression analysis are reported in Figure 2 (Regression tables are included in Appendix A). To facilitate the interpretation of the results we report normal regression coefficients and not hazard ratios.

The findings lend support to the argument that authoritarian regimes are risk-averse and take the profile of their diaspora into consideration when deciding whether to enfranchise them. As hypothesized (H1), autocracies which have a larger diaspora residing in democratic destination countries are significantly less likely to extend voting rights to emigrants. This finding also holds true when we consider the share of refugees among the population abroad in democratic residence countries (H2). Both effects are highly significant highlighting that autocrats are weary of diasporas if a significant share sits in countries who protect their freedom to associate, mobilize and voice criticism. The potential backlash from enfranchising them outweighs the benefits of repressing, co-opting or politically legitimizing them to lobby foreign governments in their favour. Noteworthy, electoral considerations regarding the potential impact of the diaspora vote seem secondary in this context. We additionally tested whether the size of the diaspora electorate relative to the resident population produces similar results, finding no effect (see Appendix A, Table 3).

In turn, power transitions have a positive effect on diaspora enfranchisement in autocracies. The effect of general regime transitions is positive but only significant on the 10% level, which leads us to reject Hypothesis 3 since this variable also fails to pass some of our robustness tests (see next section). However, successful coups are significant on the 5% level, indicating that they drive autocrats to adopt emigrant voting rights. As hypothesized (H4), this finding supports our argument that coup-imposed power transitions increase the regime’s need for legitimacy, repression and co-optation, rendering the adoption of democratic institutions in the transnational space (i.e. diaspora voting rights) more likely.

Successful coups usually bring power transitions and violence under one umbrella. To further disentangle these two factors, we also test the effect of political violence without considering power transitions. We replace the coup variable with the political violence indicator *v2caviol* from V-Dem and create a rolling five-year average to model a climate in which political violence is persistent.^116^ However, political violence is an insignificant predictor for emigrant enfranchisement (Appendix B, Tables 1 and 2). In combination with our finding that general power transitions are largely irrelevant, these results suggest that successful coups matter because illegally overthrowing the government increases the new regime’s need for legitimacy. Diaspora voting rights not only serve as a tool for co-optation but are used as a signalling tool in this context.

**Figure 3.**
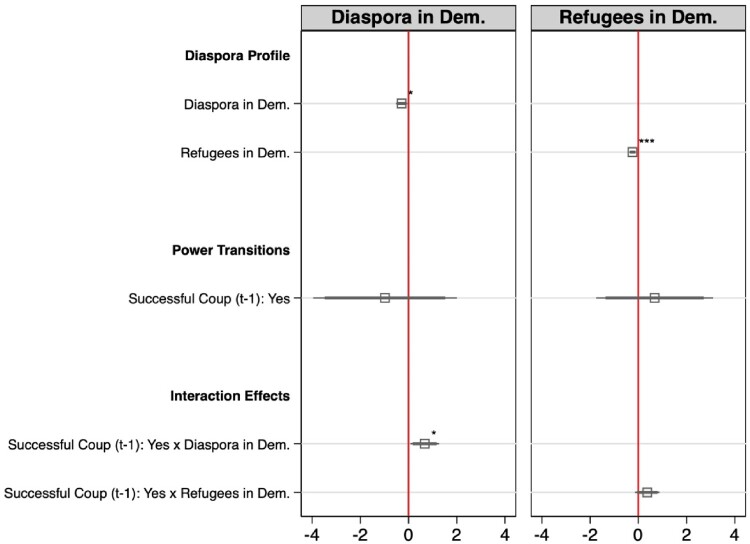
Cox regression results with interaction effects. Note: Coefficient estimates from Cox regressions with the thick bar showing the corresponding 90% confidence interval and the thin bar showing the corresponding 95% confidence interval. Dependent Variable is emigrant enfranchisement. p-values shown alongside markers: * p<0.05, ** p<0.01, *** p<0.001. All models include: state capacity (t-1), EMB capacity (t-1), GDP per capita (t-1), GDP per capita growth (t-1), time in power, civil society strength, foreign aid dependency (t-1), emigrant suffrage diffusion, regional democracy score, democratic past.

Turning to the question of how the interaction between diaspora profile and successful coups influence autocratic regimes’ decision to adopt emigrant voting rights (H4.1 & H4.2), we depart from M4 and run two separate models, each featuring one diaspora profile variable. Each model includes one individual diaspora characteristic, the successful coup dummy and the interaction of both. The results are reported in Figure 3 (see Appendix A for the regression tables).

Both models again confirm the importance of the diaspora profile as a relevant predictor for the enfranchisement of non-resident citizens in autocracies. Authoritarian regimes with a larger share of the diaspora residing in democracies and a larger share of refugees in democracies, continue to have significantly lower chances to adopt diaspora suffrage. Interestingly, successful coups in combination with a larger diaspora residing in democracies significantly increase the likelihood of diaspora enfranchisement. This could be because successful coup plotters have a high interest in legitimizing their illegal power grab. This is in line with previous research, which shows that coup plotters frame the illegal removal of the government as a commitment to restore democracy,^117^ or celebrate elections thereafter.^118^ In this regard, the increased need for these actions trumps the potential risks associated with the democratic destination effect.^119^ Additionally, the new regime might see emigrants, who have left the country under the previous regime, as supporters. Granting them voting rights the new incumbent can strengthen this connection. The interaction of successful coups and the share of refugees residing in democracies only partially supports this line of reasoning as the effect moves in the expected direction, but significance levels vary by coup coding and sample specifications (see robustness tests).

### Robustness tests

We test the robustness of the results in three different ways. First, we use the coup measurement by Powell and Thyne replacing the one by Marshall and Marshall.^120^ The results are reported in Appendix C. We are able to replicate all the main results. Second, some autocracies democratize the year after they have enfranchised their diaspora. Here, democratizing forces might have already been active when the country enacted the diaspora voting bill. To account for this scenario, we drop these regime spells. We are able to replicate all main effects (see Appendix D). The significance of the diaspora share in democratic host countries variable even increases (M1 & M4). Third, datasets differ in their conceptualization and coding of countries being an autocracy or democracy. We therefore replicate the main analysis using the V-Dem dataset’s Regimes of the World (RoW) measure for the case selection.^121^ We collapse closed autocracies and electoral autocracies as well as electoral democracies and liberal democracies to have a binary autocracy-democracy measure similar to the one by Boix and colleagues.^122^ The General Power Transition variable is coded 1 if in the previous year the head of state (*v2exnamhos*) has changed, and 0 otherwise. Regime age is measured in number of years until the head of state changes. We also conduct the previous robustness tests. Using the findings from Figures 2 and 3 as the point of departure, we are able to largely replicate all main findings, which are reported in Appendix E. A large diaspora and refugee share in democracies remain red flags for autocrats who consider enfranchising their diaspora unless a country experiences a successful coup which positively impacts the likelihood of authoritarian regimes to adopt diaspora suffrage. And while significance levels are lower, they remain consistently significant.

### Organizing elections abroad

Amongst the 41 autocracies who have extended voting rights to their diaspora between 1990 and 2010, 27 have also subsequently organized elections abroad. Our theory suggests that autocrats continue to evaluate the risks and benefits when deciding to follow up the *de jure* enfranchisement with *de facto* implementation of the first election abroad. Indeed, the *de facto* scenario presents a hard case for our theory since it requires autocracies to accept even larger risks. We focus on the first overseas election as the outcome variable, replacing the *de jure* coding with the *de facto* coding, following the approach of Wellman and colleagues.^123^ To further explore our main findings, we take Model 1–6 as a point of departure. Results are included in Appendix F.

We find that the diaspora profile still strongly matters. Both a large diaspora share in democracies and a large refugee share in democracies are significantly and negatively associated with the likelihood of *de facto* enfranchisement. This finding is robust across all specified models. General power transitions are again positive, but, as before, lack robust significance. Results are more mixed concerning the effect of coups. First, coups become insignificant in Model 4. Second, concerning the diaspora in democracies share, the interaction moves in the expected direction, but fails to reach robust significance (Model 5). Third, the coup – refugee share interaction remains positive and significant, suggesting that coups continue to trump the risks autocracies associate with a large enfranchised refugee population that resides in democracies (Model 6). However, unlike in the *de jure* analysis, this model additionally reports a negative and significant individual coup effect that supersedes the interaction effect. This means that successful coups may lower risks associated with enfranchisement in countries with large refugee populations living in democracies. But successful coups also contain high risks that in the end will discourage autocrats to organize elections abroad.

In sum, autocracies are highly risk averse and use diaspora profile characteristics as cues to assess potential backlashes resulting from both *de jure* and *de facto* enfranchisement. In post-coup contexts, *de jure* emigrant voting rights are granted to legitimize, co-opt and signal commitment to democracy. However, autocracies would not go as far as organizing elections abroad (*de facto*) which suggests that controlling and collecting information via voting is generally less important in post-coup contexts than we initially proposed. Paired with a high share of refugees that reside in democracies the likelihood of holding elections abroad even decreases significantly. Autocrats seem to value safety over potential benefits and postpone, sometimes indefinitely, *de facto* elections in these scenarios.

## Conclusion

This article set out to explore the largely overlooked phenomenon of autocratic diaspora enfranchisement. We have linked the question of why autocrats grant voting rights to their citizens abroad to three previously largely disconnected literatures dealing with electoral autocracies, emigrant enfranchisement and authoritarian sending state policies. Our core argument posits that emigrant voting rights constitute a tool for autocratic leaders to govern their diaspora and gain international legitimacy. Yet, autocrats are highly risk averse and consider two key characteristics of their diaspora when estimating the potential risks of granting them voting rights: the regime type of the destination countries and the size of the refugee population within these destination countries. We empirically substantiate these arguments by demonstrating that autocrats are less likely to enfranchise a diaspora that largely resides in democracies or consists of refugees in democracies. Autocratic homelands appear to take seriously the risk of opening up their electoral processes to overseas voters and the enfranchisement trend is tempered by the perception of “democratic diasporas”. This notion extends beyond granting the right on paper to actually holding elections abroad.

In turn, successful coups render diaspora suffrage adoption more likely. This is because after irregularly seizing power the new regime seeks international legitimacy and needs to govern, i.e. co-opt and potentially repress, its citizens abroad to cement its rule. Emigrant enfranchisement is, in other words, not just a by-product of broader processes of democratization as the existing literature on emigrant enfranchisement has often argued. In fact, our results highlight that a regime transition to democracy is not a necessary condition for the adoption of diaspora voting laws at all. Instead, we show that autocracies pass emigrant suffrage even though they remain autocratic thereafter. What greatly matters for *de jure* emigrant enfranchisement in an autocratic setting is the way in which power is shifted. In contrast, first-time *de facto* enfranchisement processes seem to be less affected by successful coups. Their effect is conditioned by the relative number of refugees living in democracies. In post-coup settings, autocrats will rarely organize transnational elections, but chances rise the more refugees live in democracies as they present a group of potential allies for the new regime.

These findings advance the understanding of emigrant voting rights that is so far dominated by a focus on democracies. We also make an important contribution to both the research field on autocratic resilience and autocratic state-diaspora relations which have paid scant attention to this issue at most. Additionally, our coup finding contributes to other work on the consequences of coups.^124^ External voting rights are a strategy employed by autocrats seeking to prolong their tenure. They aim to connect and exert control over their internationally mobile citizenry.^125^

In terms of the broader literature on autocratic resilience, external voting rights present a critical case to better understand why authoritarian states adopt democratic institutions and wish to connect with their diaspora. Future research could build on our findings and expand our theoretical framework to investigate autocrats’ approach towards passive voting rights. Building on our theoretical framework, scholars could study other fields of diaspora governance in autocracies, such as citizenship laws, economic and cultural policies. Using large-N datasets to investigate why and how autocracies engage in these policy areas would help advance the literature on autocratic state-diaspora relations, which still remains largely dominated by qualitative case studies.

## Supplementary Material

Supplemental Material

## Data Availability

The data that support the findings of this study are openly available in Figshare at https://figshare.com/s/203bac600daf6a64b39f.

## Notes

1 Wellman, Allen, and Nyblade, “The Extraterritorial Voting Rights.”

2 Collyer, “Transnational Political Participation”; Moss, “Transnational Repression.”

3 Albertus and Menaldo, “Dictators as Founding Fathers?”; Ginsburg and Simpser, *Constitutions in Authoritarian Regimes*.

4 Gandhi and Przeworski, “Authoritarian Institutions”; Magaloni, “Credible Power-Sharing”; Geddes, “What Do We Know”; Gandhi and Lust-Okar, “Elections Under Authoritarianism”; Levitsky and Way, *Competitive Authoritarianism*; Svolik, *The Politics of Authoritarian Rule*; Schedler, “The Politics of Uncertainty”; Miller, “The Autocratic Ruling Parties”; Wright and Escribà-Folch, “Authoritarian Institutions.”

5 Bush, “International Politics.”

6 For a review of the literature, see Meng, Paine, and Powell, “Authoritarian Power Sharing.”

7 Smith and Guarnizo, *Transnationalism from Below*; Collyer, *Emigration Nations*; Gamlen, *Human Geopolitics*; Østergaard-Nielsen, “The Politics of Migrants’ Transnational Political Practices”; Gamlen, “The Emigration State.”

8 Tsourapas, “Global Autocracies”; Miller and Peters, “Restraining the Huddled Masses”; Dalmasso et al., “Intervention: Extraterritorial Authoritarian Power”; Dukalskis et al., “The Long Arm”; Glasius, “Extraterritorial Authoritarian Practices.”

9 Lafleur, *Transnational Politics*; Caramani and Grotz, “Beyond Citizenship and Residence?”; Fliess and Østergaard-Nielsen, “Extension of Voting Rights”; Umpierrez de Reguero, Finn, and Peltoniemi, “Missing Links”; Wellman, Allen, and Nyblade, “The Extraterritorial Voting Rights.”

10 Brand, “Authoritarian States and Voting”; Brand, “Arab Uprisings and the Changing”; Jaulin and Nolan, “The Limits to External”; Umpierrez de Reguero, Yener-Roderburg, and Cartagena, “Political Regimes.”

11 Wall, “Indonesia.”

12 Fliess and Østergaard-Nielsen, “Extension of Voting Rights.”

13 Turcu and Urbatsch, “Diffusion of Diaspora Enfranchisement.”

14 Erlingsson and Tuman, “External Voting Rights.”

15 Turcu and Urbatsch, “European Ruling Parties’ Electoral”; Østergaard-Nielsen, Ciornei, and Lafleur, “Why Do Parties Support”; Wellman, “Emigrant Inclusion in Home”; Lafleur, *Transnational Politics*.

16 Escobar, “Extraterritorial Political Rights”; Rhodes and Harutyunyan, “Extending Citizenship to Emigrants”; Pogonyi, “Four Patterns of Non-Resident.”

17 Alemán and Woods, “No Way Out.”

18 Escribà-Folch, Meseguer, and Wright, “Remittances and Protest”; Ahmed, “The Perils of Unearned.”

19 Miller and Peters, “Restraining the Huddled Masses.”

20 Tsourapas, “Global Autocracies.”

21 Gerschewski, “The Three Pillars.”

22 Tsourapas, “Global Autocracies”; Glasius, “Extraterritorial Authoritarian Practices.”

23 Michaelsen, “Exit and Voice”; Wackenhut and Orjuela, “Engaging the next Generation.”

24 Østergaard-Nielsen, “Turkey and the ‘Euro-Turks’.”

25 Moss, Michaelsen, and Kennedy, “Going after the Family.”

26 Dukalskis et al., “The Long Arm.”

27 Gamlen, “The Emigration State.”

28 Glasius, “Extraterritorial Authoritarian Practices.”

29 Gerschewski, “The Three Pillars.”

30 Tsourapas, “Global Autocracies.”

31 Wrighton, “Authoritarian Regime Stabilization.”

32 Gerschewski, “The Three Pillars.”

33 Penadés and Velasco, “Plebiscites.”

34 Magaloni, *Voting for Autocracy*; Ginsburg and Simpser, *Constitutions in Authoritarian Regimes*.

35 Williamson, “Elections, Legitimacy, and Compliance”; Schedler, “Elections Without Democracy.”

36 Magaloni, *Voting for Autocracy*; Gandhi and Lust-Okar, “Elections Under Authoritarianism”; Svolik, *The Politics of Authoritarian Rule*; Miller, “The Autocratic Ruling Parties”; Blaydes, *Elections and Distributive Politics*.

37 Ginsburg and Simpser, *Constitutions in Authoritarian Regimes*; Schedler, “Elections Without Democracy.”

38 Brownlee, *Authoritarianism in an Age*; Magaloni, “Credible Power-Sharing.”

39 Michaelsen, “Exit and Voice.”

40 Boccagni and Ramírez, “Building Democracy or Reproducing.”

41 Turcu and Urbatsch, “European Ruling Parties’ Electoral.”

42 Brand, “Authoritarian States and Voting.”

43 Østergaard-Nielsen, “Sending Country Policies.”

44 Wellman, Allen, and Nyblade, “The Extraterritorial Voting Rights.”

45 Fliess, “Campaigning across Continents.”

46 Betts and Jones, *Mobilising the Diaspora*, 220.

47 Brand, *Citizens Abroad*; Iskander, *Creative State*; Østergaard-Nielsen, *Transnational Politics*; Lyons and Mandaville, *Politics from Afar*; Betts and Jones, *Mobilising the Diaspora*.

48 Lacy, “Host Country Issues.”

49 AfricaNews, “‘Electoral Process Badly Underway’.”

50 Cameroon Postline, “Cameroonian Diaspora Threatens Election.”

51 Bekaj and Antara, *Political Participation of Refugees*, 77.

52 Hirt and Mohammad, “The Lack of Political Space.”

53 Levitt and de la Dehesa, “Transnational Migration.”

54 Laguerre, *Diasporic Citizenship: Haitian Americans*.

55 Pérez-Armendáriz and Crow, “Do Migrants Remit Democracy?”; Kapur, “Political Effects of International Migration”; Barsbai et al., “The Effect of Labor Migration”; Batista, Seither, and Vicente, “Do Migrant Social Networks.”

56 Pérez-Armendáriz and Crow, “Do Migrants Remit Democracy?”; Levitt, “Social Remittances.”

57 Escribà-Folch, Meseguer, and Wright, “Remittances and Protest”; Peters and Miller, “Emigration and Political Contestation.”

58 Miller and Peters, “Restraining the Huddled Masses.”

59 Lafleur, “The Enfranchisement of Citizens”; Hutcheson and Arrighi, “Keeping Pandora’s (Ballot) Box”; Turcu, “Reactive Limits to Diaspora.”

60 Collyer, “Inside Out?”

61 Wellman, “Refugee Status.”

62 Brand, “Arab Uprisings and the Changing.” 60.

63 CIP Eleições, “CNE & CC Exclude.”

64 Slavu, “External Voting for Afghanistan,” 3.

65 Knutsen, Nygård, and Wig, “Autocratic Elections: Stabilizing Tool”; Bernhard, B. Edgell, and Lindberg, “Institutionalising Electoral Uncertainty.”

66 Bunce and Wolchik, “Defeating Dictators”; Donno, “Elections and Democratization.”

67 Wellman, “Refugee Status.”

68 Grace and Mooney, “Peacebuilding Through the Electoral,” 102.

69 Farr et al., “Refugees in Elections: The Liberian Experience,” 17.

70 Hirt and Mohammad, “The Lack of Political Space.”

71 Zakaryan and Antara, *Political Participation of Refugees*.

72 Gberie, “Liberia and Sierra Leone,” 210.

73 Atassi, “Huge Turnout for Syrian,” cited after Bekaj and Antara, *Political Participation of Refugees*, 77.

74 See e.g. Meng, Paine, and Powell, “Authoritarian Power Sharing.”

75 Greitens, *Dictators and Their Secret*.

76 Albertus and Menaldo, “Dictators as Founding Fathers?”; Geddes, Wright, and Frantz, *How Dictatorships Work*, 36–8.

77 Miller, “The Strategic Origins.”

78 Magaloni, *Voting for Autocracy*; Ginsburg and Simpser, *Constitutions in Authoritarian Regimes*.

79 Dietrich and Wright, “Foreign Aid Allocation Tactics”; Marinov and Goemans, “Coups and Democracy”; Williamson, “Elections, Legitimacy, and Compliance.”

80 Yukawa, Hidaka, and Kushima, “Coups and Framing.”

81 Thyne and Powell, “Coup d’état or Coup.”

82 Jaulin and Nolan, “The Limits to External,” 256.

83 Yefet, “Egypt’s Diaspora Policy,” 8.

84 Boix, Miller, and Rosato, “A Complete Data Set.”

85 Coppedge et al., “V-Dem [Country–Year/Country–Date].”

86 Cleves et al., “An Introduction to Survival.”

87 See e.g. Turcu and Urbatsch, “Diffusion of Diaspora Enfranchisement”; Wellman, Allen, and Nyblade, “The Extraterritorial Voting Rights.”

88 Wellman, Allen, and Nyblade, “The Extraterritorial Voting Rights.”

89 United Nations, Department of Economic and Social Affairs, Population Division, “International Migrant Stock 2019.”

90 Coppedge et al., “V-Dem [Country–Year/Country–Date].”

91 United Nations High Commissioner for Refugees, “Population Figures.”

92 Miller, “The Autocratic Ruling Parties.”

93 Ibid.

94 Marshall and Marshall, “Coup d’état Events.”

95 Ibid.

96 Powell and Thyne, “Global Instances of Coups.”

97 Geddes, Wright, and Frantz, *How Dictatorships Work*.

98 Singer, “Reconstructing the Correlates”; Greig and Enterline, “National Material Capabilities.”

99 Pallister, “Migrant Populations and External.”

100 Coppedge et al., “V-Dem [Country–Year/Country–Date].”

101 Gleditsch, “Expanded Trade and GDP.”

102 Ibid.

103 Magaloni, *Voting for Autocracy*.

104 Miller, “The Autocratic Ruling Parties.”

105 Escobar, “Extraterritorial Political Rights”; Brand, “Arab Uprisings and the Changing.”

106 Coppedge et al., “V-Dem [Country–Year/Country–Date].”

107 See also, Bernhard, B. Edgell, and Lindberg, “Institutionalising Electoral Uncertainty.”

108 Miller, “The Strategic Origins.”

109 AidData, “AidData Core Research Release, Version 3.1”; Gleditsch, “Expanded Trade and GDP.”

110 Caramani and Grotz, “Beyond Citizenship and Residence?”; Lafleur, “The Enfranchisement of Citizens”; Turcu and Urbatsch, “Diffusion of Diaspora Enfranchisement.”

111 See e.g. Richter and Wurster, “Policy Diffusion among Democracies.”

112 Wellman, Allen, and Nyblade, “The Extraterritorial Voting Rights.”

113 Coppedge et al., “V-Dem [Country–Year/Country–Date].”

114 Miller, “Democratic Pieces.”

115 Coppedge et al., “V-Dem [Country–Year/Country–Date].”

116 Ibid.

117 Yukawa, Hidaka, and Kushima, “Coups and Framing.”

118 Grewal and Kureshi, “How to Sell a Coup.”

119 Securing foreign aid does not seem to be a main motivation here as we find no robust effects when interacting coups and foreign aid (see Appendix B, Tables 3 and 4). Rather, it could be that the coup plotters use transnational voting rights to quickly repress and co-opt potential opponents to cement their power. Moreover, they can use transnational voting rights as a form of rapprochement with foreign governments.

120 Powell and Thyne, “Global Instances of Coups”; Marshall and Marshall, “Coup d’état Events.”

121 Coppedge et al., “V-Dem [Country–Year/Country–Date].”

122 Boix, Miller, and Rosato, “A Complete Data Set.”

123 Wellman, Allen, and Nyblade, “The Extraterritorial Voting Rights.”

124 E.g. Marinov and Goemans, “Coups and Democracy.”

125 Tsourapas, “Global Autocracies”; Glasius, “Extraterritorial Authoritarian Practices.”
