# Supplementary material for: Why do autocracies enfranchise their citizens abroad? A large-N event history analysis, 1990–2010
Source: Democratization. 2024 Aug 22;32(3):659–83. doi: 10.1080/13510347.2024.2383795 (PMC11934954; doi:10.1080/13510347.2024.2383795)
Supplement: Supplemental Material [file FDEM_A_2383795_SM7996.pdf]

Appendices A-F

Appendix A Cox regression outputs, corresponding to Figure 2. and Figure 3 ________________2
Appendix B Cox regression results with alternative conditional effects ______________________5
Appendix C Cox regression results with coup coding by Powell and Thyne (2011) for robustness tests ____7
Appendix D Cox regression results without countries who democratize after enfranchising the diaspora ____10
Appendix E Cox regression results using the V-Dem dataset for robustness tests _____________12
Appendix F Cox regression results using de facto coding ________________________________16

Appendix A Cox regression outputs, corresponding to Figure 2. and Figure 3

1. Cox regression output corresponding to Figure 2.

DV: Emigrant Enfranchisement	(M1a) Diaspora in Democracies	(M2a) Refugees in Democracies	(M3a) General Power Transitions	(M4a) Successful Coups
Diaspora in Dem.	-0.21*			
	(0.11)			
Refugees in Dem.		-0.23***		
		(0.07)		
General Power Transition (t-1): Yes			0.78+	
			(0.42)	
Successful Coup (t-1): Yes				1.26*
				(0.64)
State Capacity (t-1)	-10.61	-9.06	-16.02	-12.76
	(13.03)	(10.77)	(20.30)	(15.20)
EMB Capacity (t-1)	0.13	0.17	0.14	0.15
	(0.15)	(0.17)	(0.15)	(0.15)
GDP per capita (t-1)	0.52**	0.58**	0.46*	0.53**
	(0.18)	(0.21)	(0.19)	(0.19)
GDP per capita growth (t-1)	-0.02+	-0.01	-0.02+	-0.02*
	(0.01)	(0.01)	(0.01)	(0.01)
Years in Power	-0.05**	-0.04**	-0.04**	-0.05**
	(0.02)	(0.02)	(0.02)	(0.02)
Civil Society Strength	0.13	-0.08	-0.03	-0.15
	(0.77)	(0.83)	(0.78)	(0.80)
Foreign Aid Dependency (t-1)	0.03	0.02	0.05	0.05
	(0.09)	(0.11)	(0.10)	(0.10)
Emigrant Franchise Diffusion: Yes	0.08	-0.06	0.14	0.05
	(0.41)	(0.45)	(0.41)	(0.44)
Regional Democracy Level	0.96	0.70	0.69	0.67
	(1.68)	(1.83)	(1.77)	(1.70)
Democratic Past	-2.71*	-2.32*	-0.85	-2.83*
	(1.14)	(1.07)	(1.34)	(1.12)
Observations	1,008	911	996	967

Robust standard errors in parentheses
*** p<0.001, ** p<0.01, * p<0.05, + p<0.10
Coefficients shown, not hazard ratios

2. Cox regression output with interaction effects, corresponding to Figure 3

DV: Emigrant enfranchisement	(M5a)<br>Diaspora in Dem.	(M6a)<br>Refugees in Dem.
Diaspora in Dem.	-0.29*	
	(0.12)	
Successful Coup (t-1) x Diaspora in Dem.	0.67*	
	(0.30)	
Refugees in Dem.		-0.24***
		(0.07)
Successful Coup (t-1) x Refugees in Dem.		0.37
		(0.26)
Successful Coup (t-1): Yes	-0.98	0.67
	(1.52)	(1.24)
State Capacity (t-1)	-10.26	-9.55
	(11.79)	(11.08)
EMB Capacity (t-1)	0.14	0.21
	(0.15)	(0.17)
GDP per capita (t-1)	0.61**	0.65**
	(0.19)	(0.22)
GDP per capita growth (t-1)	-0.02*	-0.02+
	(0.01)	(0.01)
Years in Power	-0.04**	-0.04**
	(0.01)	(0.01)
Civil Society Strength	0.12	-0.31
	(0.81)	(0.87)
Foreign Aid Dependency (t-1)	0.06	0.07
	(0.10)	(0.12)
Emigrant Franchise Diffusion: Yes	-0.00	0.03
	(0.43)	(0.44)
Regional Democracy Level	1.02	0.33
	(1.68)	(1.81)
Democratic Past	-2.62*	-2.38*
	(1.09)	(1.03)
Observations	967	886

Robust standard errors in parentheses
*** p<0.001, ** p<0.01, * p<0.05, + p<0.10
Coefficients shown, not hazard ratios

3. Cox regression output exploring the potential effect of the total diaspora size

Some studies have argued that a relatively sizeable diaspora can discourage emigrant enfranchisement because political parties are afraid that emigrant votes could swamp election results and thus tip the outcome. For example, Umpierrez et al (2023) claim that sizeable diasporas in Ireland and Uruguay are a reason that voting rights have not been granted yet. For autocracies we do not find any significant relationship between emigrant enfranchisement and a countries' diaspora size, which we operationalized as the logged share of the domestic population. We run the analysis with both case selection criteria.

	Boix et al Sample		VDem Sample	
DV: Emigrant Enfranchisement	Full Sample	No Democratizers	Full Sample	No Democratizers
Total Diaspora Size	-0.02	-0.09	0.20	0.21
	(0.15)	(0.19)	(0.15)	(0.16)
State Capacity (t-1)	-14.39	-25.29	-2.57	-3.38
	(19.51)	(53.97)	(8.60)	(10.79)
EMB Capacity (t-1)	0.16	0.13	-0.14	-0.20
	(0.15)	(0.18)	(0.15)	(0.18)
GDP per capita (t-1)	0.44*	0.54*	0.17	0.16
	(0.17)	(0.22)	(0.18)	(0.20)
GDP per capita growth (t-1)	-0.02*	-0.02*	-0.01	-0.02
	(0.01)	(0.01)	(0.01)	(0.01)
Years in Power (Boix et al)	-0.05**	-0.05**		
	(0.02)	(0.02)		
Years in Power (VDem)			-0.01	-0.02
			(0.02)	(0.02)
Civil Society Strength	-0.15	-0.80	0.57	-0.08
	(0.80)	(0.86)	(0.91)	(0.90)
Foreign Aid Dependency (t-1)	0.02	0.04	-0.03	-0.02
	(0.09)	(0.11)	(0.10)	(0.11)
Emigrant Franchise Diffusion: Yes	0.07	-0.30	-0.04	-0.49
	(0.42)	(0.57)	(0.34)	(0.43)
Regional Democracy Level	0.51	1.35	0.51	0.49
	(1.71)	(2.00)	(1.38)	(1.50)
Democratic Past (Boix et al)	-2.66*	-2.25*		
	(1.13)	(1.13)		
Democratic Past (VDem)			-0.63	-0.48
			(0.65)	(0.77)
Observations	1,002	954	1,091	1,040

Robust standard errors in parentheses
*** p<0.001, ** p<0.01, * p<0.05, + p<0.10
Coefficients shown, not hazard ratios

Appendix B Cox regression results with alternative conditional effects

1. Cox regression results with political violence replacing successful coups (Boix et al Sample)

	Full Sample (Boix et al)			No Democratizers (Boix et al)		
DV: Emigrant enfranchisement	(M7a) Political Violence	(M8a) Interaction	(M9a) Interaction	(M7b) Political Violence	(M8b) Interaction	(M9b) Interaction
Diaspora in Dem.		-0.23+			-0.44**	
		(0.12)			(0.15)	
Political Violence x Diaspora in Dem.		0.08			0.07	
		(0.08)			(0.08)	
Refugees in Dem.			-0.23***			-0.25**
			(0.07)			(0.08)
Political Violence x Refugees in Dem.			0.01			-0.03
			(0.05)			(0.05)
Political Violence	-0.03	-0.24	0.10	0.05	-0.07	0.22
	(0.15)	(0.26)	(0.23)	(0.16)	(0.30)	(0.23)
State Capacity (t-1)	-13.96	-11.43	-9.76	-20.89	-14.98	-17.37
	(18.06)	(13.86)	(10.83)	(36.52)	(19.86)	(24.92)
EMB Capacity (t-1)	0.13	0.13	0.22	0.13	0.14	0.27
	(0.16)	(0.16)	(0.21)	(0.20)	(0.22)	(0.23)
GDP per capita (t-1)	0.42*	0.50**	0.60**	0.52*	0.67**	0.64*
	(0.18)	(0.18)	(0.22)	(0.23)	(0.23)	(0.25)
GDP per capita growth (t-1)	-0.02*	-0.01+	-0.01+	-0.02*	-0.02*	-0.02*
	(0.01)	(0.01)	(0.01)	(0.01)	(0.01)	(0.01)
Years in Power	-0.05**	-0.04**	-0.04*	-0.05*	-0.04*	-0.04*
	(0.02)	(0.02)	(0.02)	(0.02)	(0.02)	(0.02)
Civil Society Strength	-0.26	-0.06	-0.30	-0.85	-0.37	-0.78
	(0.78)	(0.79)	(0.85)	(0.84)	(0.87)	(0.87)
Foreign Aid Dependency (t-1)	0.03	0.02	0.02	0.04	0.03	0.09
	(0.09)	(0.09)	(0.12)	(0.11)	(0.11)	(0.13)
Emigrant Franchise Diffusion: Yes	0.16	0.18	0.03	-0.19	-0.20	-0.38
	(0.41)	(0.40)	(0.46)	(0.56)	(0.53)	(0.57)
Regional Democracy Level	0.60	0.90	0.80	1.26	1.94	0.72
	(1.65)	(1.62)	(1.79)	(1.89)	(1.86)	(2.01)
Democratic Past	-2.85*	-2.72*	-2.35*	-2.43*	-2.13+	-1.90+
	(1.14)	(1.16)	(1.11)	(1.14)	(1.16)	(0.99)
Observations	977	977	880	929	929	856

Robust standard errors in parentheses
*** p<0.001, ** p<0.01, * p<0.05, + p<0.10
Coefficients shown, not hazard ratios

2. Cox regression results with <u>political violence</u> replacing successful coups (VDem Sample)

	Full Sample (VDem)			No Democratizers (VDem)		
DV: Emigrant enfranchisement	(M7c) Political Violence	(M8c) Interaction	(M9c) Interaction	(M7d) Political Violence	(M8d) Interaction	(M9d) Interaction
Diaspora in Dem.		-0.34**			-0.45**	
		(0.13)			(0.14)	
Political Violence x Diaspora in Dem.		0.08			0.10	
		(0.08)			(0.09)	
Refugees in Dem.			-0.21**			-0.23**
			(0.07)			(0.07)
Political Violence x Refugees in Dem.			0.03			0.01
			(0.05)			(0.05)
Political Violence	0.04	-0.17	-0.02	0.08	-0.19	0.09
	(0.13)	(0.28)	(0.20)	(0.14)	(0.31)	(0.21)
State Capacity (t-1)	-6.73	-5.40	-4.04	-7.72	-5.47	-3.83
	(8.95)	(8.21)	(8.23)	(11.85)	(10.40)	(10.16)
EMB Capacity (t-1)	-0.10	-0.11	-0.10	-0.16	-0.16	-0.10
	(0.16)	(0.16)	(0.19)	(0.18)	(0.20)	(0.22)
GDP per capita (t-1)	0.22	0.33+	0.38+	0.24	0.39+	0.38+
	(0.18)	(0.19)	(0.22)	(0.20)	(0.21)	(0.23)
GDP per capita growth (t-1)	-0.01	-0.01	-0.01	-0.02	-0.02	-0.01
	(0.01)	(0.01)	(0.01)	(0.01)	(0.01)	(0.01)
Years in Power (VDem)	-0.01	-0.01	-0.02	-0.02	-0.02	-0.02
	(0.02)	(0.02)	(0.02)	(0.02)	(0.02)	(0.02)
Civil Society Strength	0.17	0.44	0.09	-0.49	-0.13	-0.53
	(0.84)	(0.87)	(0.94)	(0.86)	(0.91)	(0.94)
Foreign Aid Dependency (t-1)	-0.01	-0.02	-0.04	0.00	-0.00	0.01
	(0.09)	(0.09)	(0.11)	(0.10)	(0.10)	(0.12)
Emigrant Franchise Diffusion: Yes	0.00	-0.01	-0.15	-0.41	-0.43	-0.58
	(0.34)	(0.34)	(0.37)	(0.44)	(0.45)	(0.46)
Regional Democracy Level	1.04	1.58	1.23	1.08	1.74	0.75
	(1.39)	(1.34)	(1.43)	(1.55)	(1.49)	(1.58)
Democratic Past (VDem)	-0.80	-0.69	-0.52	-0.65	-0.41	-0.35
	(0.69)	(0.65)	(0.56)	(0.81)	(0.76)	(0.71)
Observations	1,063	1,063	956	1,012	1,012	927

Robust standard errors in parentheses
*** p<0.001, ** p<0.01, * p<0.05, + p<0.10
Coefficients shown, not hazard ratios

3. Cox regression output exploring the conditional effect of foreign aid (Boix et al Sample)

	Full Sample (Boix et al)		No Democratizers (Boix et al)	
DV: Emigrant enfranchisement	Coup Coding (M/M)	Coup Coding (P/T)	Coup Coding (M/M)	Coup Coding (P/T)
Foreign Aid Dependency (t-1)	0.04	0.02	0.07	0.03
	(0.10)	(0.10)	(0.11)	(0.11)
Successful Coup (M/M): Yes	-0.16		0.40	
	(1.74)		(1.86)	
Successful Coup (M/M) x Foreign Aid	0.35		0.28	
	(0.43)		(0.46)	
Successful Coup (P/T): Yes		0.39		0.93
		(1.71)		(1.84)
Successful Coup (P/T) x Foreign Aid		0.20		0.15
		(0.44)		(0.47)
State Capacity (t-1)	-12.40	-12.57	-18.88	-17.54
	(14.50)	(15.56)	(29.27)	(27.09)
EMB Capacity (t-1)	0.14	0.15	0.14	0.11
	(0.15)	(0.15)	(0.19)	(0.19)
GDP per capita (t-1)	0.53**	0.45*	0.62**	0.54*
	(0.19)	(0.18)	(0.22)	(0.23)
GDP per capita growth (t-1)	-0.02*	-0.01+	-0.02*	-0.02+
	(0.01)	(0.01)	(0.01)	(0.01)
Years in Power	-0.05**	-0.05**	-0.05**	-0.05**
	(0.02)	(0.02)	(0.02)	(0.02)
Civil Society Strength	-0.12	-0.15	-0.72	-0.75
	(0.81)	(0.78)	(0.85)	(0.84)
Emigrant Franchise Diffusion: Yes	0.05	0.17	-0.20	-0.14
	(0.44)	(0.42)	(0.56)	(0.56)
Regional Democracy Level	0.76	0.53	1.25	1.19
	(1.69)	(1.72)	(1.91)	(1.98)
Democratic Past	-2.80*	-2.79*	-2.47*	-2.34*
	(1.13)	(1.13)	(1.10)	(1.10)
Observations	967	1,008	939	960

Robust standard errors in parentheses
*** p<0.001, ** p<0.01, * p<0.05, + p<0.10
Coefficients shown, not hazard ratios

4. Cox regression output exploring the conditional effect of foreign aid (VDem Sample)

	Full Sample (VDem)		No Democratizers (VDem)	
DV: Emigrant enfranchisement	Coup Coding (M/M)	Coup Coding (P/T)	Coup Coding (M/M)	Coup Coding (P/T)
Foreign Aid Dependency (t-1)	-0.01	-0.02	0.02	-0.00
	(0.09)	(0.09)	(0.10)	(0.10)
Successful Coup (M/M): Yes	-1.77		-1.15	
	(1.50)		(1.56)	
Successful Coup (M/M) x Foreign Aid	0.74*		0.63+	
	(0.36)		(0.37)	
Successful Coup (P/T): Yes		-1.39		-0.57
		(1.72)		(1.75)
Successful Coup (P/T) x Foreign Aid		0.65		0.50
		(0.42)		(0.42)
State Capacity (t-1)	-6.11	-6.55	-7.55	-7.39
	(8.64)	(8.75)	(11.55)	(11.43)
EMB Capacity (t-1)	-0.13	-0.12	-0.17	-0.19
	(0.16)	(0.15)	(0.17)	(0.17)
GDP per capita (t-1)	0.28	0.23	0.29	0.24
	(0.18)	(0.17)	(0.19)	(0.20)
GDP per capita growth (t-1)	-0.02+	-0.01	-0.02	-0.02
	(0.01)	(0.01)	(0.01)	(0.01)
Years in Power (VDem)	-0.01	-0.01	-0.02	-0.02
	(0.02)	(0.02)	(0.02)	(0.02)
Civil Society Strength	0.33	0.31	-0.35	-0.34
	(0.85)	(0.82)	(0.84)	(0.82)
Emigrant Franchise Diffusion: Yes	-0.12	0.00	-0.47	-0.42
	(0.36)	(0.34)	(0.45)	(0.44)
Regional Democracy Level	1.40	1.26	1.27	1.30
	(1.46)	(1.47)	(1.63)	(1.67)
Democratic Past (VDem)	-0.79	-0.79	-0.71	-0.64
	(0.71)	(0.70)	(0.84)	(0.83)
Observations	1,052	1,094	1,021	1,043

Robust standard errors in parentheses
*** p<0.001, ** p<0.01, * p<0.05, + p<0.10
Coefficients shown, not hazard ratios

Appendix C Cox regression results with coup coding by Powell and Thyne (2011) for robustness tests

DV: Emigrant Enfranchisement	(M4b) Successful Coups	(M5b) Diaspora in Democracies	(M6b) Refugees in Democracies
Diaspora in Dem.		-0.29*	
		(0.12)	
Successful Coup (P/T) x Diaspora in Dem.		0.68*	
		(0.29)	
Refugees in Dem.			-0.25***
			(0.07)
Successful Coup (P/T) x Refugees in Dem.			0.41+
			(0.23)
Successful Coup (P/T): Yes	1.21+	-1.06	0.66
	(0.65)	(1.49)	(1.13)
State Capacity (t-1)	-12.74	-10.02	-8.83
	(16.00)	(11.97)	(10.60)
EMB Capacity (t-1)	0.16	0.16	0.19
	(0.15)	(0.15)	(0.17)
GDP per capita (t-1)	0.45*	0.54**	0.60**
	(0.18)	(0.18)	(0.22)
GDP per capita growth (t-1)	-0.01+	-0.02*	-0.01
	(0.01)	(0.01)	(0.01)
Years in Power	-0.05**	-0.04**	-0.04**
	(0.02)	(0.01)	(0.01)
Civil Society Strength	-0.17	0.04	-0.31
	(0.77)	(0.78)	(0.85)
Foreign Aid Dependency (t-1)	0.02	0.03	0.04
	(0.10)	(0.10)	(0.12)
Emigrant Franchise Diffusion: Yes	0.17	0.11	0.08
	(0.42)	(0.42)	(0.45)
Regional Democracy Level	0.47	0.81	0.29
	(1.73)	(1.68)	(1.86)
Democratic Past	-2.80*	-2.55*	-2.21*
	(1.12)	(1.11)	(1.00)
Observations	1,008	1,008	911

Robust standard errors in parentheses
*** p<0.001, ** p<0.01, * p<0.05, + p<0.10
Coefficients shown, not hazard ratios

Appendix D Cox regression results without countries who democratize after enfranchising the diaspora

1. Models 1-4, corresponding to Figure 2.

DV: Emigrant Enfranchisement	(M1b) Diaspora in Democracies	(M2b) Refugees in Democracies	(M3b) Gen. Pow. Transit.	(M4c) Successful Coups
Diaspora in Dem.	-0.37**			
	(0.12)			
Refugees in Dem.		-0.24**		
		(0.08)		
General Power Transition (t-1): Yes			0.82+	
			(0.47)	
Successful Coup (t-1): Yes				1.54*
				(0.69)
State Capacity (t-1)	-13.83	-16.09	-22.10	-19.92
	(18.86)	(25.97)	(38.80)	(32.45)
EMB Capacity (t-1)	0.09	0.16	0.11	0.15
	(0.19)	(0.19)	(0.19)	(0.18)
GDP per capita (t-1)	0.63**	0.60**	0.52*	0.61**
	(0.21)	(0.22)	(0.23)	(0.22)
GDP per capita growth (t-1)	-0.02+	-0.02+	-0.02+	-0.02*
	(0.01)	(0.01)	(0.01)	(0.01)
Years in Power	-0.04*	-0.04*	-0.04*	-0.05**
	(0.02)	(0.02)	(0.02)	(0.02)
Civil Society Strength	-0.21	-0.45	-0.66	-0.75
	(0.85)	(0.87)	(0.82)	(0.84)
Foreign Aid Dependency (t-1)	0.04	0.07	0.06	0.07
	(0.11)	(0.12)	(0.11)	(0.11)
Emigrant Franchise Diffusion: Yes	-0.30	-0.42	-0.20	-0.20
	(0.54)	(0.57)	(0.57)	(0.56)
Regional Democracy Level	1.86	0.48	1.13	1.17
	(1.90)	(2.04)	(2.00)	(1.92)
Democratic Past	-2.14+	-1.93*	-0.65	-2.50*
	(1.12)	(0.97)	(1.37)	(1.09)
Observations	960	887	948	939

Robust standard errors in parentheses
*** p<0.001, ** p<0.01, * p<0.05, + p<0.10
Coefficients shown, not hazard ratios

2. Models 5 & 6 corresponding to Figure 3. and Models 4 – 6 using the coup coding by Powell and Thyne (2011) for robustness tests

DV: Emigrant Enfranchisement	(M5c) Diaspora in Democracies	(M6c) Refugees in Democracies	(M4d) Successful Coups	(M5d) Diaspora in Democracies	(M6d) Refugees in Democracies
Diaspora in Dem.	-0.48***			-0.52***	
	(0.14)			(0.15)	
Refugees in Dem.		-0.25**			-0.26**
		(0.08)			(0.08)
Successful Coup (M/M): Yes	-1.13	1.23			
	(1.57)	(1.38)			
Successful Coup (M/M) x Diasp. in Dem.	0.82**				
	(0.31)				
Successful Coup (M/M) x Ref. in Dem.		0.26			
		(0.30)			
Successful Coup (P/T): Yes			1.53*	-1.57	0.80
			(0.72)	(1.51)	(1.15)
Successful Coup (P/T) x Diasp. in Dem.				0.97***	
				(0.29)	
Successful Coup (P/T) x Ref. in Dem.					0.44+
					(0.24)
State Capacity (t-1)	-15.10	-17.46	-18.05	-14.35	-17.19
	(20.04)	(28.79)	(28.63)	(19.50)	(28.94)
EMB Capacity (t-1)	0.13	0.19	0.11	0.12	0.19
	(0.18)	(0.18)	(0.19)	(0.19)	(0.19)
GDP per capita (t-1)	0.74***	0.72**	0.54*	0.66**	0.63**
	(0.22)	(0.23)	(0.23)	(0.22)	(0.24)
GDP per capita growth (t-1)	-0.02+	-0.02+	-0.02+	-0.02*	-0.02+
	(0.01)	(0.01)	(0.01)	(0.01)	(0.01)
Years in Power	-0.04**	-0.04**	-0.05**	-0.04*	-0.04*
	(0.02)	(0.01)	(0.02)	(0.02)	(0.01)
Civil Society Strength	-0.31	-0.66	-0.78	-0.44	-0.78
	(0.88)	(0.90)	(0.83)	(0.86)	(0.90)
Foreign Aid Dependency (t-1)	0.08	0.12	0.03	0.05	0.08
	(0.11)	(0.13)	(0.11)	(0.11)	(0.13)
Emigrant Franchise Diffusion: Yes	-0.29	-0.31	-0.14	-0.30	-0.25
	(0.55)	(0.56)	(0.56)	(0.57)	(0.57)
Regional Democracy Level	1.72	0.26	1.14	1.50	0.03
	(1.89)	(2.01)	(1.99)	(1.93)	(2.07)
Democratic Past	-2.16*	-2.07*	-2.34*	-1.86+	-1.80*
	(1.03)	(0.92)	(1.10)	(1.05)	(0.89)
Observations	939	866	960	960	887

Robust standard errors in parentheses
*** p<0.001, ** p<0.01, * p<0.05, + p<0.10
Coefficients shown, not hazard ratios

Appendix E Cox regression results using the V-Dem dataset for robustness tests

1. Replication of Model 1 – 4

DV: Emigrant Enfranchisement	(M1c) Diaspora in Democracies	(M2c) Refugees in Democracies	(M3c) Gen. Pow. Transitions	(M4e) Successful Coups
Diaspora in Dem.	-0.30*			
	(0.12)			
Refugees in Dem.		-0.21**		
		(0.07)		
Power Transition (t-1) (VDem): Yes			-0.15	
			(0.49)	
Successful Coup (t-1): Yes				1.03+
				(0.60)
State Capacity (t-1)	-6.16	-4.55	-7.14	-6.21
	(8.31)	(8.26)	(8.96)	(8.81)
EMB Capacity (t-1)	-0.13	-0.12	-0.11	-0.11
	(0.15)	(0.17)	(0.15)	(0.15)
GDP per capita (t-1)	0.32+	0.37+	0.22	0.26
	(0.18)	(0.20)	(0.17)	(0.17)
GDP per capita growth (t-1)	-0.01	-0.01	-0.02	-0.02+
	(0.01)	(0.01)	(0.01)	(0.01)
Years in Power (VDem)	-0.01	-0.02	-0.02	-0.01
	(0.02)	(0.02)	(0.02)	(0.02)
Civil Society Strength	0.65	0.28	0.29	0.27
	(0.84)	(0.87)	(0.81)	(0.82)
Foreign Aid Dependency (t-1)	0.00	-0.01	-0.01	0.00
	(0.09)	(0.11)	(0.09)	(0.09)
Emigrant Franchise Diffusion: Yes	-0.08	-0.25	-0.05	-0.12
	(0.34)	(0.37)	(0.34)	(0.35)
Regional Democracy Level	1.79	1.37	1.11	1.13
	(1.43)	(1.48)	(1.44)	(1.44)
Democratic Past (VDem)	-0.83	-0.59	-0.87	-0.83
	(0.66)	(0.57)	(0.70)	(0.72)
Observations	1,094	987	1,094	1,052

Robust standard errors in parentheses
*** $p<0.001$, ** $p<0.01$, * $p<0.05$, + $p<0.10$
Coefficients shown, not hazard ratios

2. Replication of Model 5 & 6 using once with the coup coding by Marshall and Marshall (2019) and once with the coup coding by Powell and Thyne (2011)

DV: Emigrant Enfranchisement	(M5e) Diaspora in Democracies	(M6e) Refugees in Democracies	(M4f) Successful Coups	(M5f) Diaspora in Democracies	(M6f) Refugees in Democracies
Diaspora in Dem.	-0.36**			-0.36**	
	(0.13)			(0.13)	
Refugees in Dem.		-0.22**			-0.23***
		(0.07)			(0.07)
Successful Coup (M/M): Yes	-1.36	0.25			
	(1.63)	(0.91)			
Successful Coup (M/M) x Diasp. in Dem.	0.67+				
	(0.39)				
Successful Coup (M/M) x Ref. in Dem.		0.35			
		(0.22)			
Successful Coup (P/T): Yes			1.11+	-1.49	0.27
			(0.62)	(1.63)	(0.93)
Successful Coup (P/T) x Diasp. in Dem				0.75+	
				(0.39)	
Successful Coup (P/T) x Ref. in Dem.					0.40+
					(0.22)
State Capacity (t-1)	-4.97	-3.73	-6.67	-5.59	-4.01
	(8.23)	(8.29)	(8.91)	(8.18)	(8.28)
EMB Capacity (t-1)	-0.13	-0.08	-0.10	-0.09	-0.09
	(0.16)	(0.16)	(0.15)	(0.15)	(0.17)
GDP per capita (t-1)	0.35+	0.37+	0.23	0.33+	0.39+
	(0.18)	(0.21)	(0.17)	(0.18)	(0.21)
GDP per capita growth (t-1)	-0.02	-0.01	-0.01	-0.01	-0.01
	(0.01)	(0.01)	(0.01)	(0.01)	(0.01)
Years in Power (VDem)	-0.01	-0.01	-0.01	-0.01	-0.02
	(0.02)	(0.02)	(0.02)	(0.02)	(0.02)
Civil Society Strength	0.64	0.07	0.24	0.57	0.07
	(0.87)	(0.88)	(0.79)	(0.84)	(0.87)
Foreign Aid Dependency (t-1)	0.01	-0.00	-0.01	0.00	-0.00
	(0.10)	(0.11)	(0.09)	(0.09)	(0.11)
Emigrant Franchise Diffusion: Yes	-0.21	-0.24	-0.01	-0.12	-0.23
	(0.37)	(0.36)	(0.34)	(0.35)	(0.36)
Regional Democracy Level	1.65	0.89	1.02	1.58	1.05
	(1.45)	(1.48)	(1.44)	(1.44)	(1.50)
Democratic Past (VDem)	-0.78	-0.52	-0.79	-0.72	-0.46
	(0.70)	(0.59)	(0.71)	(0.67)	(0.57)
Observations	1,052	961	1,094	1,094	987

Robust standard errors in parentheses
*** p<0.001, ** p<0.01, * p<0.05, + p<0.10
Coefficients shown, not hazard ratios

3. Replication of Model 1 – 4 without countries who democratize after enfranchising the diaspora

DV: Emigrant Enfranchisement	(M1d) Diaspora in Democracies	(M2d) Refugees in Democracies	(M3d) Gen. Pow. Transit.	(M4g) Successful Coups
Diaspora in Dem.	-0.38**			
	(0.13)			
Refugees in Dem.		-0.23**		
		(0.07)		
Power Transition (t-1) (VDem): Yes			-0.14	
			(0.54)	
Successful Coup (t-1): Yes				1.28*
				(0.60)
State Capacity (t-1)	-6.70	-4.44	-8.27	-7.66
	(10.60)	(10.21)	(12.01)	(11.86)
EMB Capacity (t-1)	-0.21	-0.16	-0.18	-0.16
	(0.17)	(0.18)	(0.17)	(0.17)
GDP per capita (t-1)	0.36+	0.36+	0.22	0.28
	(0.20)	(0.21)	(0.19)	(0.19)
GDP per capita growth (t-1)	-0.02	-0.01	-0.02	-0.02
	(0.01)	(0.01)	(0.01)	(0.01)
Years in Power (VDem)	-0.02	-0.03	-0.03	-0.02
	(0.02)	(0.02)	(0.02)	(0.02)
Civil Society Strength	0.14	-0.28	-0.34	-0.37
	(0.85)	(0.86)	(0.82)	(0.81)
Foreign Aid Dependency (t-1)	0.02	0.03	0.01	0.03
	(0.10)	(0.12)	(0.10)	(0.10)
Emigrant Franchise Diffusion: Yes	-0.52	-0.67	-0.48	-0.45
	(0.44)	(0.46)	(0.44)	(0.44)
Regional Democracy Level	2.02	0.86	1.14	1.03
	(1.62)	(1.64)	(1.62)	(1.61)
Democratic Past (VDem)	-0.63	-0.49	-0.76	-0.76
	(0.79)	(0.72)	(0.84)	(0.85)
Observations	1,043	958	1,043	1,021

Robust standard errors in parentheses
*** p<0.001, ** p<0.01, * p<0.05, + p<0.10
Coefficients shown, not hazard ratios

4. Replication of Model 4 – 6 without countries who democratize after enfranchising the diaspora, using once the coup coding by Marshall and Marshall (2019) and once the coup coding by Powell and Thyne (2011)

DV: Emigrant Enfranchisement	(M5g) Diaspora in Democracies	(M6g) Refugees in Democracies	(M4h) Successful Coups	(M5h) Diaspora in Democracies	(M6h) Refugees in Democracies
Diaspora in Dem.	-0.46***			-0.48***	
	(0.14)			(0.15)	
Refugees in Dem.		-0.24**			-0.25***
		(0.08)			(0.07)
Successful Coup (M/M): Yes	-1.70	0.41			
	(1.61)	(0.89)			
Successful Coup (M/M) x Diasp. in Dem.	0.86*				
	(0.39)				
Successful Coup (M/M) x Refug. in Dem.		0.40+			
		(0.21)			
Successful Coup (P/T): Yes			1.39*	-1.90	0.38
			(0.64)	(1.56)	(0.94)
Successful Coup (P/T) x Diasp. in Dem.				0.98**	
				(0.37)	
Successful Coup (P/T) x Refug. in Dem.					0.48*
					(0.22)
State Capacity (t-1)	-5.70	-3.68	-7.49	-5.59	-3.63
	(10.58)	(10.25)	(11.66)	(10.26)	(10.20)
EMB Capacity (t-1)	-0.18	-0.14	-0.17	-0.16	-0.12
	(0.17)	(0.18)	(0.17)	(0.18)	(0.18)
GDP per capita (t-1)	0.41*	0.44*	0.24	0.38+	0.39+
	(0.20)	(0.22)	(0.19)	(0.20)	(0.22)
GDP per capita growth (t-1)	-0.02	-0.01	-0.02	-0.02	-0.01
	(0.01)	(0.01)	(0.01)	(0.01)	(0.01)
Years in Power (VDem)	-0.02	-0.02	-0.02	-0.02	-0.02
	(0.02)	(0.02)	(0.02)	(0.02)	(0.02)
Civil Society Strength	0.04	-0.48	-0.38	-0.04	-0.59
	(0.89)	(0.90)	(0.81)	(0.88)	(0.89)
Foreign Aid Dependency (t-1)	0.04	0.06	0.01	0.03	0.03
	(0.11)	(0.12)	(0.10)	(0.10)	(0.12)
Emigrant Franchise Diffusion: Yes	-0.57	-0.64	-0.42	-0.59	-0.63
	(0.47)	(0.45)	(0.44)	(0.48)	(0.45)
Regional Democracy Level	1.68	0.57	1.10	1.71	0.56
	(1.64)	(1.67)	(1.65)	(1.64)	(1.69)
Democratic Past (VDem)	-0.59	-0.43	-0.65	-0.42	-0.30
	(0.83)	(0.74)	(0.83)	(0.80)	(0.73)
Observations	1,021	936	1,043	1,043	958

Robust standard errors in parentheses
*** p<0.001, ** p<0.01, * p<0.05, + p<0.10
Coefficients shown, not hazard ratios

Appendix F Cox regression results using *de facto* coding

1. Replication of Model 1- 4 using *de facto* coding, Full Sample (**Boix et al**)

DV: Emigrant Enfranchisement (*de facto*)	(M1e) Diaspora in Democracies	(M2e) Refugees in Democracies	(M3e) General Power Transitions	(M4h) Successful Coups
Diaspora in Dem.	-0.37*			
	(0.16)			
Refugees in Dem.		-0.23**		
		(0.09)		
General Power Transition (t-1): Yes			1.07*	
			(0.48)	
Successful Coup (t-1): Yes				0.96
				(0.71)
State Capacity (t-1)	-44.41	-51.79	-60.66	-59.20
	(74.16)	(72.94)	(69.89)	(74.99)
EMB Capacity (t-1)	0.36*	0.35+	0.40*	0.43*
	(0.18)	(0.18)	(0.17)	(0.18)
GDP per capita (t-1)	0.30	0.27	0.26	0.19
	(0.26)	(0.26)	(0.25)	(0.28)
GDP per capita growth (t-1)	0.01	0.01+	0.01	0.01
	(0.01)	(0.01)	(0.01)	(0.01)
Years in Power	-0.04*	-0.04**	-0.03*	-0.04*
	(0.02)	(0.02)	(0.02)	(0.02)
Civil Society Strength	-0.62	-0.87	-1.34	-1.37
	(1.05)	(0.96)	(0.95)	(0.95)
Foreign Aid Dependency (t-1)	-0.01	-0.07	0.02	-0.00
	(0.12)	(0.12)	(0.11)	(0.12)
Emigrant Franchise Diff. (*de facto*): Yes	0.24	0.20	0.36	0.17
	(0.38)	(0.37)	(0.38)	(0.37)
Regional Democracy Level	1.13	1.16	1.80	1.01
	(1.82)	(1.72)	(1.76)	(1.75)
Democratic Past	-1.19	-1.16	-0.11	-1.45
	(0.91)	(0.88)	(1.13)	(0.91)
Observations	1,231	1,129	1,215	1,190

Robust standard errors in parentheses
*** p<0.001, ** p<0.01, * p<0.05, + p<0.10
Coefficients shown, not hazard ratios

2. Replication of Model 5 & 6 using *de facto* coding, Full Sample (**Boix et al**)

DV: Emigrant Enfranchisement (*de facto*)	(M5i)	(M6i)	(M5j)	(M6j)
Diaspora in Dem.	-0.40*		-0.40*	
	(0.17)		(0.17)	
Refugees in Dem.		-0.25**		-0.24**
		(0.09)		(0.09)
Successful Coup (M/M): Yes	-7.58	-6.52***		
	(10.13)	(1.37)		
Successful Coup (M/M) x Diasp. in Dem.	2.08			
	(2.36)			
Successful Coup (M/M) x Refug. in Dem.		1.75***		
		(0.28)		
Successful Coup (P/T): Yes			-3.51	-6.22***
			(2.28)	(1.39)
Successful Coup (P/T) x Diasp. in Dem.			1.12*	
			(0.55)	
Successful Coup (P/T) x Refug. in Dem.				1.67***
				(0.30)
State Capacity (t-1)	-42.74	-47.85	-39.44	-46.03
	(72.12)	(70.65)	(70.24)	(70.44)
EMB Capacity (t-1)	0.38*	0.36*	0.36*	0.33+
	(0.18)	(0.17)	(0.18)	(0.18)
GDP per capita (t-1)	0.32	0.29	0.30	0.28
	(0.29)	(0.28)	(0.27)	(0.26)
GDP per capita growth (t-1)	0.01	0.01	0.01	0.01
	(0.01)	(0.01)	(0.01)	(0.01)
Years in Power	-0.03*	-0.04**	-0.04*	-0.04**
	(0.02)	(0.01)	(0.02)	(0.01)
Civil Society Strength	-0.67	-1.00	-0.61	-0.88
	(1.10)	(1.00)	(1.06)	(0.97)
Foreign Aid Dependency (t-1)	0.02	-0.04	-0.01	-0.07
	(0.13)	(0.13)	(0.12)	(0.12)
Emigrant Franchise Diff. (*de facto*): Yes	0.11	0.09	0.23	0.23
	(0.40)	(0.38)	(0.38)	(0.37)
Regional Democracy Level	1.14	1.03	1.17	1.16
	(1.81)	(1.69)	(1.84)	(1.71)
Democratic Past	-1.14	-1.13	-1.14	-1.10
	(0.92)	(0.89)	(0.90)	(0.88)
Observations	1,190	1,104	1,231	1,129

Robust standard errors in parentheses
*** p<0.001, ** p<0.01, * p<0.05, + p<0.10
Coefficients shown, not hazard ratios

3. Replication of Model 1- 4 using *de facto* coding, No Democratizer Sample (**Boix et al**)

DV: Emigrant Enfranchisement (*de facto*)	(M1f)	(M2f)	(M3f)	(M4i)
Diaspora in Dem.	-0.45**			
	(0.16)			
Refugees in Dem.		-0.27**		
		(0.09)		
General Power Transition (t-1): Yes			1.00*	
			(0.49)	
Successful Coup (t-1): Yes				0.98
				(0.70)
State Capacity (t-1)	-85.45	-88.19	-97.79	-102.11
	(83.82)	(78.96)	(77.30)	(80.36)
EMB Capacity (t-1)	0.36+	0.35+	0.42*	0.42*
	(0.20)	(0.18)	(0.19)	(0.20)
GDP per capita (t-1)	0.32	0.28	0.25	0.22
	(0.29)	(0.28)	(0.28)	(0.29)
GDP per capita growth (t-1)	0.01	0.01	0.01	0.01
	(0.01)	(0.01)	(0.01)	(0.01)
Years in Power	-0.03*	-0.03**	-0.03+	-0.03*
	(0.01)	(0.01)	(0.01)	(0.01)
Civil Society Strength	-0.62	-0.94	-1.48	-1.30
	(1.09)	(1.01)	(0.99)	(0.99)
Foreign Aid Dependency (t-1)	0.04	-0.02	0.06	0.04
	(0.13)	(0.13)	(0.12)	(0.12)
Emigrant Franchise Diff (*de facto*): Yes	-0.03	-0.07	0.11	0.02
	(0.40)	(0.38)	(0.40)	(0.38)
Regional Democracy Level	0.96	0.81	1.51	0.99
	(1.98)	(1.85)	(1.83)	(1.88)
Democratic Past	-0.95	-0.95	0.09	-1.34
	(0.88)	(0.82)	(1.12)	(0.89)
Observations	1,211	1,124	1,195	1,189

Robust standard errors in parentheses
*** p<0.001, ** p<0.01, * p<0.05, + p<0.10
Coefficients shown, not hazard ratios

4. Replication of Model 5 & 6 using *de facto* coding, No Democratizer Sample (**Boix et al**)

DV: Emigrant Enfranchisement (*de facto*)	(1)<br>(M5j)	(2)<br>(M6j)	(3)<br>(M5k)	(4)<br>(M6k)
Diaspora in Dem.	-0.48**		-0.49**	
	(0.16)		(0.16)	
Refugees in Dem.		-0.28**		-0.29**
		(0.09)		(0.09)
Successful Coup (M/M): Yes	-7.86	-6.64***		
	(11.08)	(1.42)		
Successful Coup (M/M) x Diasp. in Dem.	2.17			
	(2.59)			
Successful Coup (M/M) x Refug. in Dem.		1.78***		
		(0.30)		
Successful Coup (P/T): Yes			-3.68+	-6.46***
			(2.21)	(1.43)
Successful Coup (P/T) x Diasp. in Dem.			1.18*	
			(0.54)	
Successful Coup (P/T) x Refug. in Dem.				1.73***
				(0.32)
State Capacity (t-1)	-84.15	-85.55	-79.29	-80.79
	(83.73)	(78.39)	(83.50)	(77.74)
EMB Capacity (t-1)	0.34+	0.33+	0.36+	0.33+
	(0.20)	(0.18)	(0.20)	(0.18)
GDP per capita (t-1)	0.38	0.34	0.32	0.29
	(0.30)	(0.29)	(0.29)	(0.28)
GDP per capita growth (t-1)	0.01	0.01	0.01	0.01
	(0.01)	(0.01)	(0.01)	(0.01)
Years in Power	-0.03*	-0.03**	-0.03*	-0.03**
	(0.01)	(0.01)	(0.01)	(0.01)
Civil Society Strength	-0.47	-0.86	-0.63	-0.97
	(1.15)	(1.04)	(1.11)	(1.02)
Foreign Aid Dependency (t-1)	0.07	0.01	0.05	-0.02
	(0.14)	(0.14)	(0.13)	(0.13)
Emigrant Franchise Diff. (*de facto*): Yes	-0.07	-0.07	-0.04	-0.03
	(0.39)	(0.38)	(0.40)	(0.39)
Regional Democracy Level	1.16	0.90	0.99	0.81
	(2.00)	(1.85)	(2.01)	(1.85)
Democratic Past	-0.95	-0.97	-0.90	-0.90
	(0.88)	(0.83)	(0.87)	(0.82)
Observations	1,189	1,103	1,211	1,124

Robust standard errors in parentheses
*** p<0.001, ** p<0.01, * p<0.05, + p<0.10
Coefficients shown, not hazard ratios

5. Replication of Model 1- 4 using *de facto* coding, Full Sample (**VDem**)

DV: Emigrant Enfranchisement (*de facto*)	(M1g)	(M2g)	(M3g)	(M4j)
Diaspora in Dem.	-0.47**			
	(0.14)			
Refugees in Dem.		-0.21**		
		(0.08)		
Power Transition (t-1) (VDem): Yes			0.24	
			(0.45)	
Successful Coup (t-1): Yes				0.71
				(0.96)
State Capacity (t-1)	-7.81	-8.02	-10.09	-9.84
	(9.48)	(9.58)	(10.76)	(10.76)
EMB Capacity (t-1)	0.06	0.06	0.10	0.12
	(0.15)	(0.15)	(0.15)	(0.15)
GDP per capita (t-1)	0.36	0.33	0.23	0.25
	(0.23)	(0.24)	(0.22)	(0.23)
GDP per capita growth (t-1)	0.01	0.01	0.01	0.01
	(0.01)	(0.01)	(0.01)	(0.01)
Years in Power (VDem)	-0.04	-0.03	-0.03	-0.03
	(0.02)	(0.02)	(0.02)	(0.02)
Civil Society Strength	-0.07	-0.51	-0.80	-0.83
	(0.95)	(0.92)	(0.86)	(0.89)
Foreign Aid Dependency (t-1)	0.06	0.01	0.05	0.07
	(0.11)	(0.12)	(0.10)	(0.10)
Emigrant Franchise Diff (*de facto*): Yes	0.29	0.34	0.41	0.29
	(0.34)	(0.33)	(0.32)	(0.33)
Regional Democracy Level	2.66+	2.61+	2.15	2.02
	(1.50)	(1.46)	(1.45)	(1.44)
Democratic Legacy (VDem): Yes	-0.81	-0.77	-1.03	-1.05
	(0.88)	(0.86)	(0.91)	(0.91)
Observations	1,315	1,203	1,315	1,273

Robust standard errors in parentheses
*** p<0.001, ** p<0.01, * p<0.05, + p<0.10
Coefficients shown, not hazard ratios

6. Replication of Model 5 & 6 using *de facto* coding, Full Sample (**VDem**)

DV: Emigrant Enfranchisement (*de facto*)	(M5l)	(M6l)	(M5m)	(M6m)
Diaspora in Dem.	-0.49***		-0.49**	
	(0.15)		(0.15)	
Refugees in Dem.		-0.23**		-0.20*
		(0.08)		(0.08)
Successful Coup (t-1): Yes	-10.04	-15.21**		
	(8.02)	(5.72)		
Successful Coup (M/M): Yes	2.63			
	(1.91)			
Successful Coup (M/M) x Diasp. in Dem.		3.63**		
		(1.27)		
Successful Coup (M/M) x Refug. in Dem.			0.54	1.33
			(1.19)	(0.83)
Successful Coup (P/T): Yes			0.36	
			(0.27)	
Successful Coup (P/T) x Diasp. in Dem.				0.02
				(0.23)
State Capacity (t-1)	-7.12	-7.69	-7.40	-8.01
	(9.53)	(9.50)	(9.24)	(9.49)
EMB Capacity (t-1)	0.09	0.09	0.09	0.09
	(0.15)	(0.15)	(0.15)	(0.16)
GDP per capita (t-1)	0.36	0.32	0.38	0.33
	(0.24)	(0.25)	(0.23)	(0.24)
GDP per capita growth (t-1)	0.01	0.01	0.01	0.01
	(0.01)	(0.01)	(0.01)	(0.01)
Years in Power (VDem)	-0.03	-0.03	-0.03	-0.03
	(0.02)	(0.02)	(0.02)	(0.02)
Civil Society Strength	-0.11	-0.62	-0.08	-0.56
	(0.98)	(0.95)	(0.97)	(0.92)
Foreign Aid Dependency (t-1)	0.07	0.02	0.06	0.01
	(0.12)	(0.13)	(0.11)	(0.12)
Emigrant Franchise Diff. (*de facto*): Yes	0.16	0.24	0.29	0.34
	(0.35)	(0.35)	(0.34)	(0.33)
Regional Democracy Level	2.36	2.35+	2.65+	2.63+
	(1.48)	(1.43)	(1.53)	(1.49)
Democratic Past (VDem)	-0.85	-0.78	-0.76	-0.74
	(0.91)	(0.87)	(0.88)	(0.87)
Observations	1,273	1,177	1,315	1,203

Robust standard errors in parentheses
*** p<0.001, ** p<0.01, * p<0.05, + p<0.10
Coefficients shown, not hazard ratios

7. Replication of Model 1- 4 using *de facto* coding, No Democratizer Sample (**VDem**)

DV: Emigrant Enfranchisement (*de facto*)	(M1h)	(M2h)	(M3h)	(M4k)
Diaspora in Dem.	-0.57***			
	(0.17)			
Refugees in Dem.		-0.22*		
		(0.09)		
Power Transition (t-1) (VDem): Yes			0.03	
			(0.53)	
Successful Coup (t-1): Yes				1.22
				(0.92)
State Capacity (t-1)	-5.77	-7.40	-8.89	-8.94
	(10.05)	(10.25)	(11.23)	(11.11)
EMB Capacity (t-1)	0.07	0.09	0.11	0.12
	(0.18)	(0.17)	(0.18)	(0.17)
GDP per capita (t-1)	0.42+	0.34	0.29	0.37
	(0.25)	(0.26)	(0.24)	(0.25)
GDP per capita growth (t-1)	0.01	0.01	0.00	0.00
	(0.01)	(0.01)	(0.01)	(0.01)
Years in Power (VDem)	-0.02	-0.02	-0.02	-0.02
	(0.02)	(0.02)	(0.02)	(0.02)
Civil Society Strength	-0.26	-0.85	-1.11	-1.04
	(1.08)	(0.99)	(0.95)	(0.96)
Foreign Aid Dependency (t-1)	0.10	0.05	0.09	0.12
	(0.13)	(0.13)	(0.12)	(0.12)
Emigrant Franchise Diff. (*de facto*): Yes	0.09	0.15	0.22	0.17
	(0.36)	(0.35)	(0.35)	(0.35)
Regional Democracy Level	2.52	2.34	2.22	2.29
	(1.72)	(1.60)	(1.61)	(1.62)
Democratic Past (VDem)	-0.36	-0.48	-0.70	-0.78
	(0.89)	(0.88)	(0.91)	(0.92)
Observations	1,282	1,188	1,282	1,259

Robust standard errors in parentheses
*** p<0.001, ** p<0.01, * p<0.05, + p<0.10
Coefficients shown, not hazard ratios

8. Replication of Model 5 & 6 using *de facto* coding, No Democratizer Sample (**VDem**)

DV: Emigrant Enfranchisement (*de facto*)	(M5n)	(M6n)	(M5o)	(M6o)
Diaspora in Dem.	-0.59***		-0.60***	
	(0.17)		(0.17)	
Refugees in Dem.		-0.23*		-0.23**
		(0.09)		(0.09)
Successful Coup (M/M): Yes	-21.32	-7.74***		
	(15.85)	(2.16)		
Successful Coup (M/M) x Diasp. in Dem.	5.39			
	(3.68)			
Successful Coup (M/M) x Refug. in Dem.		2.11***		
		(0.42)		
Successful Coup (P/T): Yes			-6.70	-7.56***
			(4.93)	(2.15)
Successful Coup (P/T) x Diasp. in Dem.			2.00+	
			(1.14)	
Successful Coup (P/T) x Refug. in Dem.				2.10***
				(0.43)
State Capacity (t-1)	-5.67	-7.19	-5.37	-6.95
	(9.86)	(10.19)	(9.86)	(10.14)
EMB Capacity (t-1)	0.07	0.08	0.08	0.09
	(0.17)	(0.16)	(0.18)	(0.17)
GDP per capita (t-1)	0.48+	0.42	0.43	0.36
	(0.27)	(0.27)	(0.26)	(0.26)
GDP per capita growth (t-1)	0.00	0.00	0.01	0.01
	(0.01)	(0.01)	(0.01)	(0.01)
Years in Power (VDem)	-0.02	-0.02	-0.02	-0.02
	(0.02)	(0.02)	(0.02)	(0.02)
Civil Society Strength	-0.09	-0.84	-0.28	-0.91
	(1.12)	(1.01)	(1.10)	(1.01)
Foreign Aid Dependency (t-1)	0.12	0.08	0.10	0.05
	(0.14)	(0.14)	(0.13)	(0.14)
Emigrant Franchise Diff. (*de facto*): Yes	0.04	0.12	0.08	0.19
	(0.36)	(0.36)	(0.36)	(0.36)
Regional Democracy Level	2.66	2.37	2.52	2.33
	(1.73)	(1.59)	(1.75)	(1.60)
Democratic Past (VDem)	-0.54	-0.57	-0.37	-0.49
	(0.94)	(0.89)	(0.90)	(0.88)
Observations	1,259	1,166	1,282	1,188

Robust standard errors in parentheses
*** p<0.001, ** p<0.01, * p<0.05, + p<0.10
Coefficients shown, not hazard ratios
